# The Mottling Phenotype in Chickens Shows Genetic Heterogeneity and Is Caused by Mutations at the *EDNRB2* Locus

**DOI:** 10.1002/age.70168

**Published:** 2026-07-17

**Authors:** Jingyi Li, Chiara Bortoluzzi, McKaela Hodge, Bertrand Bed'hom, Yifan Guo, Brian Davis, Benjamin Dorshorst, Paul Siegel, David Gourichon, Michèle Tixier‐Boichard, Leif Andersson

**Affiliations:** ^1^ Key Laboratory of Agricultural Animal Genetics, Breeding, and Reproduction of Ministry of Education, College of Animal Science and Technology Huazhong Agricultural University Wuhan China; ^2^ Department of Veterinary Integrative Biosciences, College of Veterinary Medicine and Biomedical Sciences Texas A&M University College Station Texas USA; ^3^ Department of Animal and Poultry Sciences Virginia Polytechnic Institute and State University Blacksburg Virginia USA; ^4^ Department of Animal Sciences, Animal Breeding and Genomics Wageningen University & Research Gelderland the Netherlands; ^5^ INRAE, AgroParisTech, GABI University Paris‐Saclay Jouy‐en‐Josas France; ^6^ ISYEB, MNHN, CNRS, Sorbonne Université, EPHE Université des Antilles Paris France; ^7^ INRAE PEAT Nouzilly France; ^8^ Science for Life Laboratory, Department of Medical Biochemistry and Microbiology Uppsala University Uppsala Sweden

## Abstract

Mottling is a widespread intra‐feather pattern present in many breeds of domestic chickens worldwide and inherited as an autosomal recessive trait. An amino acid substitution Arg332His in the *EDNRB2* gene was previously reported as responsible for the mottling phenotype in several Japanese breeds of chickens. Here, we show that this substitution is not causal for mottling in Mottled Houdan chicken, a breed that originated in France. A strong association between a 230 kb region harboring the *EDNRB2* gene and the mottling phenotype was documented by linkage mapping. After the screening of SNPs, indels, and structural variants in this region via whole genome sequencing, a missense mutation Ala228Thr of *EDNRB2* showed complete association in Mottled Houdan, and three other mottling breeds. It was also associated with the mottled phenotype in an experimental chicken line segregating for the phenotype. Furthermore, we documented significant allelic imbalance of *EDNRB2* in heterozygous birds, implying that regulatory mutation(s) may also contribute to the mottling phenotype. However, no single mutation showed perfect concordance with the mottling phenotype in all mottled breeds, providing evidence for genetic heterogeneity at this locus. We propose that there are at least three different alleles responsible for mottling in chickens. Our results provide an example of parallel evolution of mottling alleles in different breeds of domestic chicken.

## 1 Introduction

**FIGURE 1:**
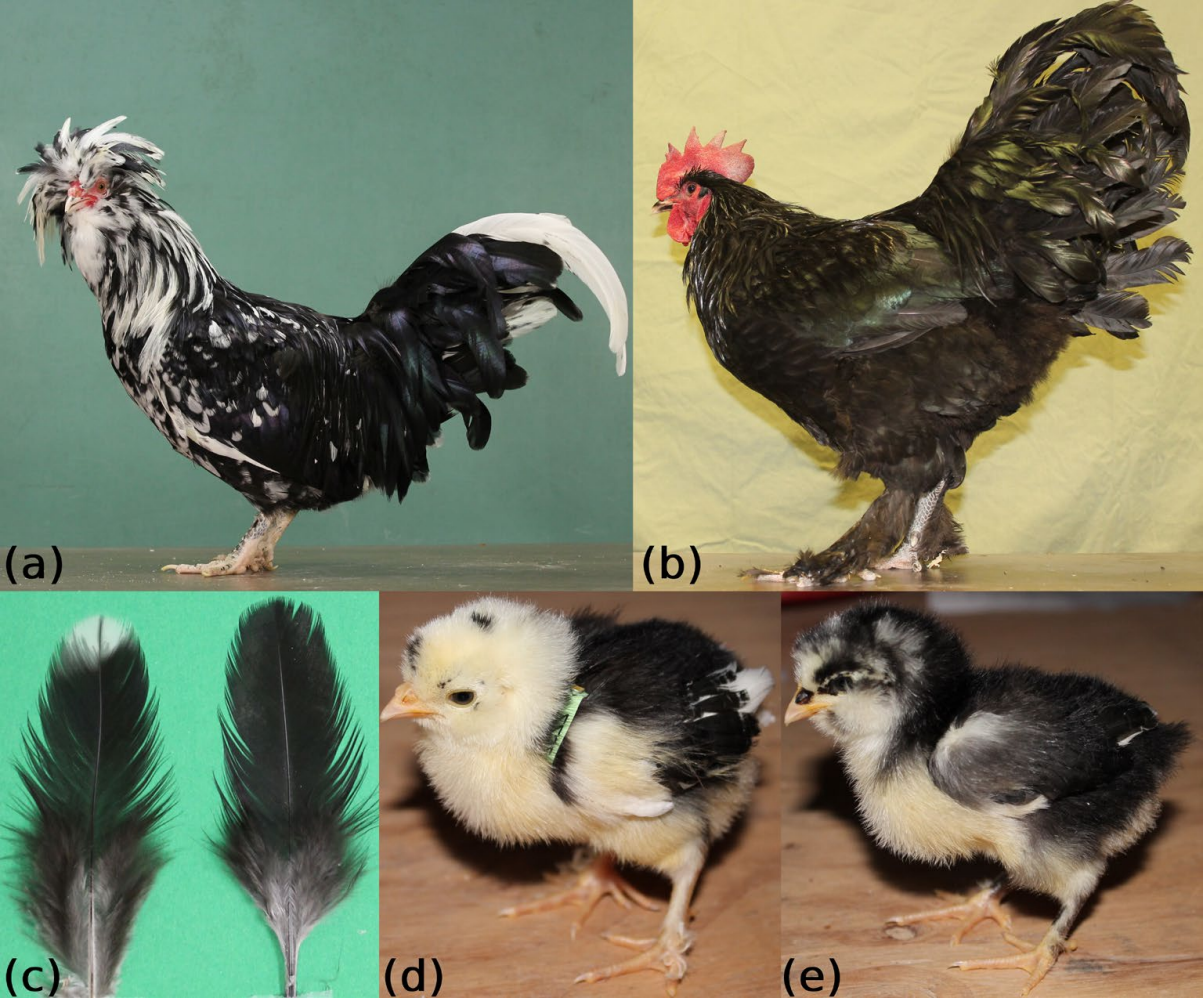
Pictures of chickens with or without mottling on black background, photo by Jingyi Li. (a) Mottled Houdan male (b) Non‐mottled Black Langshan male (c) Adult feathers collected from a mottled Houdan hen (left) and a Black Langshan hen (right) (d) down pattern of a mottled chick (e) down pattern of a non‐mottled chick.

Domesticated chickens provide excellent materials for the study of pigmentation and pattern formation. The development of melanocytes in chicken can be divided into two major stages: migration and differentiation from neural crest cells to the dermis, and from the lower budge of feather follicle to the feather vane (Inaba and Chuong 2020). Mottling is a common intra‐feather pattern in domestic chickens. In black background plumage, mottling expresses as black feathers with white distal tips (Figure 1a–c). Black mottled chicks also show restricted ventral eumelanin (Figure 1d,e). Thus, plumage patterns at two different ages are affected.

The mottling mutation (*MO*) is inherited as an autosomal recessive allele (Asmundson and Milne 1930; Somes Jr. 1980). The occurrence of large patches of white feathers had been attributed to another putative locus called *PI* (for pied), but it was soon demonstrated that *MO* and *PI* were actually two alleles at the same locus (Carefoot 1987), with symbol *MO*. Grafting experiments showed that the mottling down pattern was caused by retarded migration of melanoblasts during early development (Schaible 1968). Early differentiation of melanoblasts may cause slow proliferation and retarded migration of melanocytes in mottled chickens. A reduced rate of migration may also delay the arrival of melanocytes so that the tip of the feather becomes unpigmented (Schaible 1968).

By cross breeding, Somes Jr. (1980) has shown that *MO* in black mottled chickens (Mottled Houdan) is allelic to the mutation contributing to tricolored patterns in other breeds, i.e., speckling, mille fleur, porcelain, and spangled. Therefore, tricolored patterns are considered as a subtype of mottling, in which *MO* is assumed to generate a white distal tip followed by a V‐shaped black band on the feather vane. Thus, the color of the rest of the feather is independent of *MO*, which could be black (black mottled) or brown (tricolored) (Somes Jr. 1980).

Kinoshita et al. (2014) reported an allelism between MO*MO and tyrosinase‐independent recessive white (MO*W) in Japanese chickens; the wild‐type allele is denoted MO*N. Miwa et al. (2007) identified an Arg332His amino acid change in the endothelin receptor B2 (*EDNRB2*) gene that is assumed to be causal for the panda phenotype in Japanese quail, a phenotype similar to MO*W in chickens. Based on the plumage phenotype of an F_1_ hybrid between a panda quail and a MO*W chicken, Kinoshita et al. (2014) concluded that mottling was caused by a mutation in *EDNRB2*. They reported that a Cys244Phe amino acid substitution in *EDNRB2* was associated with the MO*W allele, while Arg332His in the same gene was associated with the MO*MO allele in Japanese chickens. In ducks, the spot pattern is also associated with an Arg332His substitution in *EDNRB2* (Li et al. 2015). Thus, four phenotypes in these three avian species have been shown to be associated with *EDNRB2* mutations, and three of the four involve exactly the same amino acid substitution, Arg332His. Besides, a frame‐shift mutation caused by a 14‐bp insertion of *EDNRB2* results in white plumage in the swan geese (Ouyang et al. 2022; Yang et al. 2022); three missense mutations and a 37‐kb tandem duplication involving *EDNRB2* were also found to be associated with either white or regional white plumage in the domestic pigeon (Maclary et al. 2023; Mao et al. 2024).

*EDNRB2* encodes a G protein‐coupled receptor that together with its ligand, endothelin 3 (EDN3), plays a prominent role in melanoblast differentiation and migration (Harris et al. 2008). Vertebrates generally possess three types of endothelin receptor genes, *EDNRA*, *EDNRB1*, and *EDNRB2* as a result of whole‐genome duplication events that occurred in their common ancestors. In mammals, however, *EDNRB2* was lost secondarily from a chromosome that gave rise to the sex chromosomes (Braasch et al. 2009). *EDNRB2* in birds is exclusively responsible for pigmentation, while *EDNRB1* in birds and *EDNRB* in mammals are in charge of peripheral nervous system development and thus relatively conserved. Consequently, numerous *EDNRB2* variants affecting pigmentation variation in birds have been identified (Maclary and Shapiro 2025), as described above. Thus, the avian *EDNRB2* gene is less constrained than the single mammalian *EDNRB* gene affecting development of both melanocytes and the peripheral nervous system.

In our study, the initial goal was to identify the causal mutation of the *MO* allele in Mottled Houdan (Figure 1a), a breed that originated in France, as the reported missense mutation Arg332His was not detected in this breed. Here we show that another missense mutation (Ala228Thr) and down‐regulation of *EDNRB2* in feather follicles is associated with mottling in the Houdan breed. However, these missense mutations are not associated with mottling across all breeds, providing evidence that mottling is genetically heterogeneous in chickens.

## 2 Results

### 2.1 Assignment of *MO* to the *EDNRB2* Genomic Region Using SNP Analysis and Linkage Mapping

**FIGURE 2:**
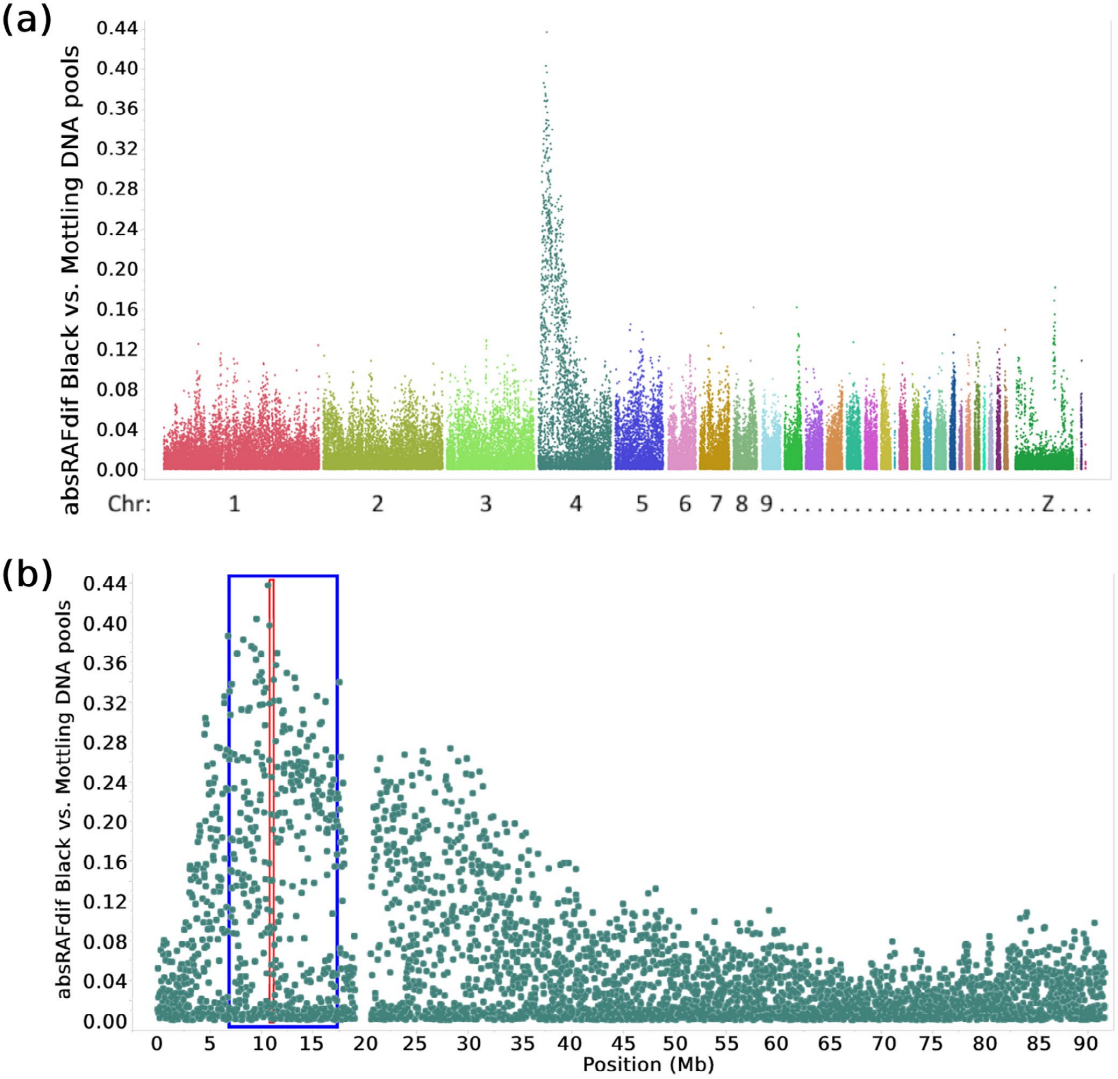
Genetic mapping of *MO* using a Houdan × Langshan backcross. (a) Genome‐wide absRAFdif values calculated using a 60 K SNP chip and DNA pools representing mottled (MO*MO/*MO*) and black (MO*MO/*N*) progeny. (b) The same absRAFdif values for SNPs on chromosome 4. SNPs located within the 10.80 Mb region (blue boxed area) gave absRAFdif values above 0.34. Red boxed area indicates the 230 kb linkage mapping region, harboring the *MO* mutation.

A Mottled Houdan (MO*MO/*MO*) x Black Langshan (MO*N/*N*) backcross family was built. The number of mottled (MO*N/*N*) and black (MO*MO/*N*) backcross individuals (113 and 108, respectively) did not differ from the expected 1:1 ratio (*p* = 0.74). A genome‐wide screen using a 60 K SNP chip and the two pooled samples of mottling and black progeny revealed one obvious peak of high absRAFdif (absolute relative allelic frequency differences) values on chromosome 4 (Figure 2a). A 10.80 Mb region from 6.86 to 17.66 Mb (genome coordinates according to the GalGal6 assembly) contained all SNPs with absRAFdif values greater than 0.34 (Figure 2b).

**FIGURE 3:**
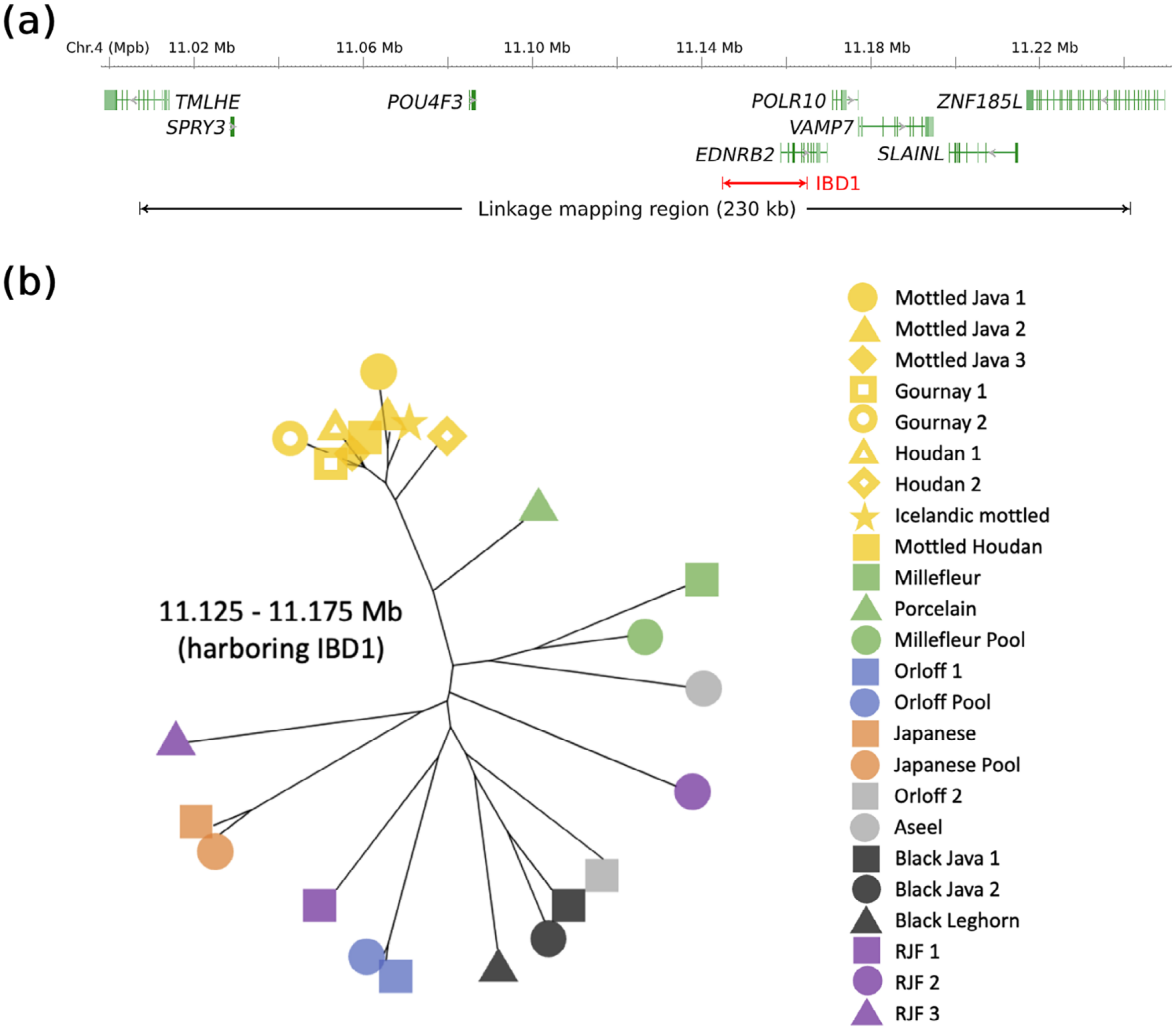
IBD region shared by nine non‐Japanese black mottled samples representing four breeds (yellow labels). (a) Genomic contents of the linkage mapping region for *MO* in Houdan. Identical‐by‐descent region 1 (IBD1) shared by European black mottled breeds and Mottled Java, is highlighted by red. (b) Genetic distance trees based on pair‐wise genetic distances (50 kb window) harboring IBD1.

Within this region, eight SNPs which all had relatively high absRAFdif values were selected for the first round of linkage mapping using all the individuals in the mapping population. As a result of this analysis, *MO* was mapped to a 0.9 Mb region defined by rs16360752 (10.71 Mb) and rs317734713 (11.60 Mb) (Table 1). In a second round of linkage mapping, nine additional SNPs were selected as markers based on whole genome sequence (WGS) data from the parental lines of the backcross family. Within the 0.9 Mb region, the candidate region was further narrowed down to a 230 kb interval, between rs736157261 (11.01 Mb) and rs313821755 (11.24 Mb) containing *EDNRB2* and seven other genes (Table 1, Figures 2b and 3a).

**TABLE 1:** Linkage mapping of *MO* in a Mottled Houdan × Black Langshan backcross using individual SNP genotyping.

dbSNP (reference allele/non‐reference allele)	Frequency of non‐reference allele according to WGS data		absRAFdif according to 60 K SNP chip data^a^	Position on Chr. 4 (bp, GalGal6)	Genetic distance from *MO* (cM)	LOD score^b^
	Parents: Mottled Houdan	Parents: Black Langshan				
rs14424110 (A/G)	1.00	0.00	0.39	6 863 063	9.9	34.3
rs314222717 (A/C)	1.00	0.00	0.38	8 322 758	7.7	40.5
rs315977835 (A/G)	0.83	0.00	0.40	9 597 996	5.2	32.7
rs16360752 (C/T)	0.00	1.00	0.44	10 705 613	2.3	56.2
rs313406325 (A/G)	0.00	1.00	—	10 856 646	0.5	63.7
rs736157261 (C/T)	0.00	1.00	—	11 007 003	0.5	63.7
rs16361338 (A/G)	0.00	1.00	—	11 122 775	0.0	66.5
rs317986873 (G/T)	1.00	0.00	—	11 172 708	0.0	66.5
rs314405946 (G/A)	1.00	0.00	—	11 198 832	0.0	66.5
rs313821755 (C/T)	0.00	1.00	—	11 241 378	1.8	57.8
rs16361521 (C/T)	0.00	1.00	—	11 275 557	1.8	57.8
rs15494340 (A/G)	1.00	0.00	—	11 311 842	2.3	56.2
rs313834381 (G/A)	1.00	0.00	—	11 461 125	2.3	56.2
rs317734713 (C/T)	0.00	0.97	0.37	11 602 895	2.7	54.6
rs316670962 (C/T)	0.00	0.63	0.35	12 547 722	8.4	35.5
rs14430948 (G/A)	0.00	0.85	0.34	13 309 078	9.0	35.5
rs14433304 (G/A)	0.00	0.74	0.33	15 467 425	13.1	29.2

^a^ SNPs without absRAFdif (for the second round of linkage mapping) were chosen from WGS data rather than the 60 K SNP chip data.

^b^ LOD score > 3.0 are considered genome‐wide significant.

### 2.2 Identification of Sequence Variants Associated With *MO* in Non‐Japanese Black Mottled Breeds

We next used previously reported WGS data from samples of nine mottled chicken breeds: Mottled Houdan, Gournay, Red Mottled Aseel, Red Spangled Orloff, Red Porcelain Booted Bantam, Mille Fleur Booted Bantam, Black Mottled Japanese Bantam, Black Mottled Java, and an Icelandic indigenous chicken with black mottled plumage (Table S1) to search for identical‐by‐descent (IBD) regions expected to harbor one or more causal mutations. Pair‐wise genetic distances in 50 kb windows were calculated for the 230 kb region on chromosome 4 defined by linkage mapping. As a result, no single IBD region nor any single candidate mutation shared among all 18 samples representing nine breeds was detected. This finding, combined with our clear evidence that the causal mutation for mottling in the Houdan breed must be located within the 230 kb region, implies that mottling shows genetic heterogeneity and that there is more than one mutant allele causing a very similar phenotype.

Therefore, pair‐wise genetic distances among those 18 mottled samples and 6 non‐mottled samples were used to construct genetic distance trees, aiming to identify subsets of mottled samples with shared IBD regions. Rather than the 230 kb region, a larger region was investigated to search for putative IBD regions. In the region of Chr4: 9.0–11.6 Mb, 103 genetic distance trees were built for each of the 50 kb window in a 25 kb step manner. According to the genetic distance trees, the 18 mottling samples were grouped into 6 subsets as they show sequence similarities in some regions (Table 2). We first focused on the subset of non‐Japanese black mottled breeds (i.e., European black mottled breeds including Houdan, Gournay, and Icelandic native breed, and Black Mottled Java, *n* = 9) which showed a putative IBD region defined as 11.145–11.165 Mb (IBD1, Table 2, Figure 3). Within this 20 kb region, 11 sequence variants (Table S2) match the criteria of candidate mutations for *MO*, i.e., homozygous mutant in a subset of mottling populations. rs313943998 in exon 5 of *EDNRB2* is the only coding variant, and it is the only one with a conservation score higher than 1 (PhyloP = 6.26), also the only one for which the reference allele is fixed in the 195 non‐mottling samples. Therefore, Ala228Thr in *EDNRB2* is the top candidate causal mutation in IBD1 and thus is a likely causative mutation for mottling phenotypes in non‐Japanese black mottled breeds including Mottled Houdan, i.e., the mottling breeds sharing the IBD1 region (Table 3).

**TABLE 2:** Summary of subsets of mottling samples sharing putative IBD regions in the vicinity of *EDNRB2* according to the genetic distance trees.

Subset name	Samples in the subset	IBD regions
Non‐Japanese black mottled	3 Mottled Houdan, 2 Gournay, 3 Mottled Java, and 1 Icelandic mottled	IBD1 (11.145–11.165 Mb)
Tricolored Booted Bantam	2 Mille Fleur Booted Bantam and 1 Red Porcelain Booted Bantam	IBD2 (9.100–9.200 Mb) IBD3 (9.500–10.800 Mb) IBD4 (11.200–11.300 Mb) IBD5 (11.455–11.500 Mb)
Japanese black mottled Bantam	2 Japanese black mottled Bantam	Larger than 10.700–11.600 Mb^a^
Red Spangled Orloff_1	2 Red Spangled Orloff	Larger than 10.700–11.600 Mb^a^
Red Spangled Orloff_2	1 Red Spangled Orloff	—
Red Mottled Aseel	1 Red Mottled Aseel	—

^a^ The entire first round linkage mapping region.

**TABLE 3:** A minimum of three *mottling* alleles and their top candidate mutations.^a^

Breed	Number of samples	Phenotype	Candidate mutation, position on Chr. 4 (bp, GalGal6)
Houdan	20	Black Mottled	11 164 253 Ala228Thr of *EDNRB2*
Gournay	2		
Icelandic native breed	1		
Java	3		
Japanese mottled breeds	2	Black Mottled	11 166 001 Arg332His of *EDNRB2*
Mille Fleur Booted Bantam	2	Tricolored	None
Red Porcelain Booted Bantam	1		
Red Spangled Orloff	3		
Red Mottled Aseel	1		
Mille Fleur D'Uccle Belgian	1		
Spangled Old English	1		
Speckled Sussex	1		

^a^ Details can be found in Table S2.

**FIGURE 4:**
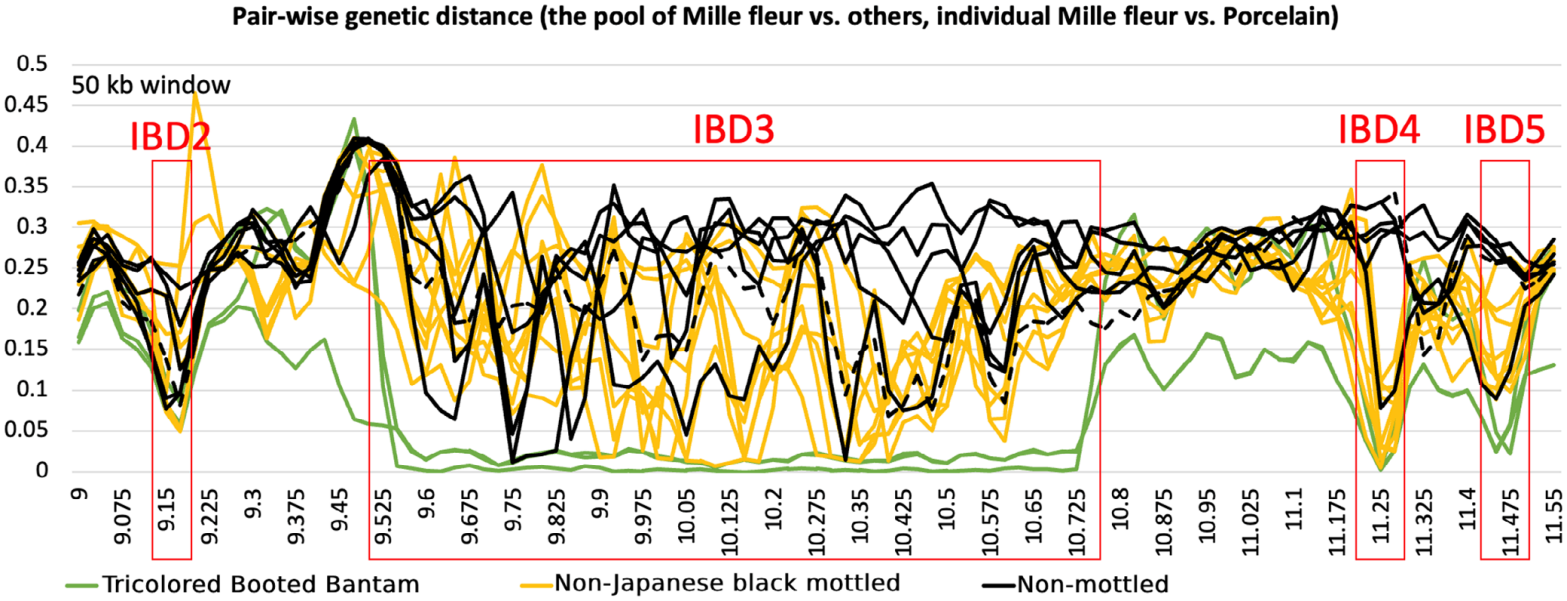
IBD regions shared by three tricolored Booted Bantam samples representing two breeds (Mille fleur, Porcelain). Plots of pair‐wise genetic distances, based on variable sites only, between the pool of Mille fleur sample and other mottled or non‐mottled samples, plus the contrast between the individual samples with Mille fleur and Red Porcelain. Green lines indicate the contrast within tricolored Booted Bantam samples (2 Mille fleur and 1 Red Porcelain), yellow lines indicate the contrast with non‐Japanese black mottled samples (3 Houdan, 2 Gournay, 3 Java, and 1 Icelandic native chicken), black lines indicate the contrast with non‐mottled samples (2 Black Java, 1 Black Leghorn, and 3 red junglefowl). The X‐axis is the genomic position on chromosome 4 in Mb. The red boxes indicate the IBD regions.

Because the gene variants underlying mottling in Houdan and tricolored breeds have been shown to be allelic by pedigree analysis (Somes Jr. 1980), but apparently not identical at the molecular level, the causal mutation(s) for mottling in tricolored breeds should also be in the vicinity of *EDNRB2*. Four putative IBD regions shared by the three tricolored Booted Bantam samples were identified based on the construction of genetic distance trees (IBD2 to 5, Table 2, Figure 4). In these regions, a total of 12 SNPs were identified as candidate mutations for *MO* in the three Booted Bantam samples. However, none of them showed strong evidence for being functionally important (Table S2).

For each of the other four subsets of samples (2 Japanese black mottled Bantam, 2 Red Spangled Orloff, another Red Spangled Orloff, and 1 pool of Red Mottled Aseel, Table 2), no shared IBD segment was found (Figures S1–S4). Thus, there are insufficient data in our dataset to identify candidate regions or mutations for *MO* in those breeds.

### 2.3 Diagnostic Tests

The *EDNRB2* Ala228Thr missense mutation was genotyped in two experimental populations, 221 individuals from the Mottled Houdan (MO*MO/*MO*) × Black Langshan (MO*N/*N*) family used for linkage mapping, and 18 individuals from an INRAE line segregating for mottling. The Ala228Thr SNP showed complete co‐segregation with the *MO* locus in both populations. The size of white spots varied considerably among birds homozygous for the Ala228Thr mutation in the INRAE line (Figure S5). Diagnostic SNP tests for Ala228Thr and the previously described Arg332His mutation were also carried out using a breed panel including 5 mottling and 68 non‐mottling chicken populations (Table S3). Among 237 non‐mottling chickens, no homozygote for the Ala228Thr was found, but 6 heterozygotes were present. The Ala228Thr missense mutation was homozygous in 17 Mottled Houdan samples but absent in the 3 tricolored individuals (Table 3, Table S3). All these birds were fixed for the wild‐type allele of Arg332His, as previously reported to be responsible for mottling in Japanese chickens (Kinoshita et al. 2014).

### 2.4 Differential Expression of *EDNRB2* Alleles Implies the Presence of *cis*‐Regulatory Mutations

**FIGURE 5:**
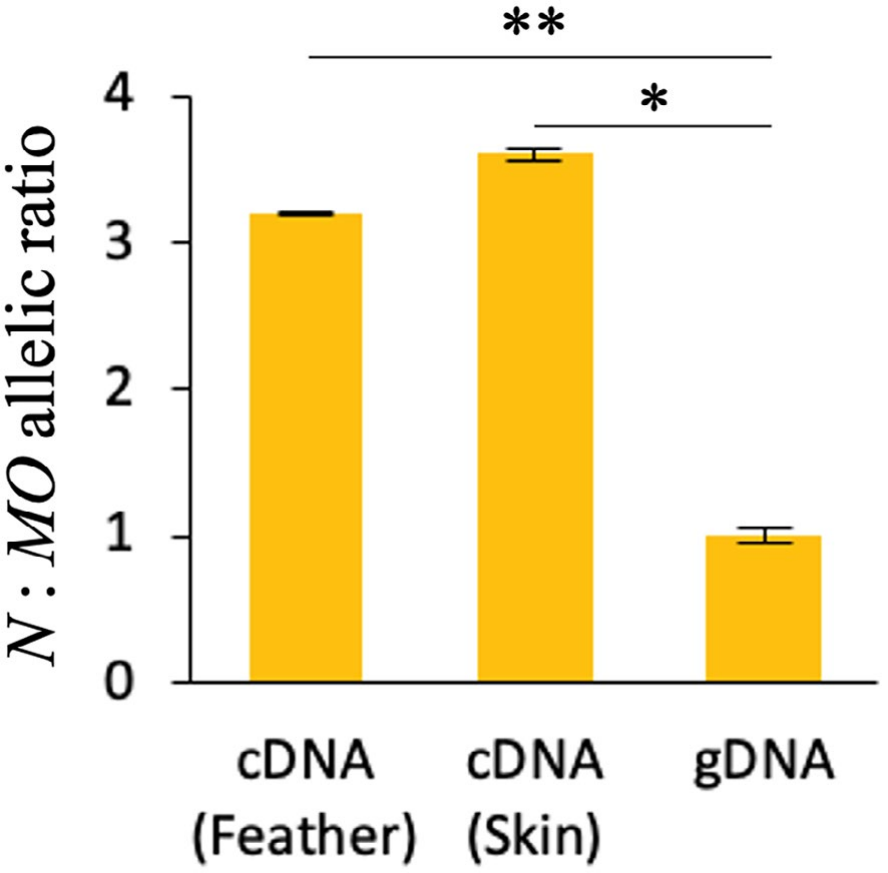
Allelic imbalance test for *EDNRB2* in two MO*MO/*N* heterozygotes. Allelic ratio of Ala228Thr was quantified using the PeakPicker 2 software. The y‐axis shows the ratio of the *N* allele over the *MO* allele in cDNA (from feather or skin tissues) or genomic DNA (gDNA). Data are represented as mean ± SEM. An asterisk signifies a significant difference (*p* < 0.05) under paired t‐test, while double asterisks indicate highly significant differences (*p* < 0.01).

A mating between Mottled Houdan (MO*MO/MO) and Black Silkie (MO*N/N) was made for an allelic imbalance experiment to test if regulatory mutations may contribute to the mottling phenotype. Sanger sequencing confirmed that the two F_1_ individuals from this mating, are heterozygous for the Ala228Thr mutation in *EDNRB2*. Therefore, this sequence polymorphism was used as marker to test for the possible presence of allelic imbalance in the expression of *EDNRB2* mRNA. The wild‐type allele showed about a three‐fold higher expression than the *MO* allele in developing feather follicles and about 3.5‐fold higher expression in skin (Figure 5). These results provide strong evidence for the presence of one or more *cis*‐acting regulatory mutations causing reduced expression of *EDNRB2* in Mottled Houdan chickens, because if *trans*‐regulatory mutations are responsible for differential expression of *EDNRB2*, the two alleles should be affected to the same degree, i.e., without allelic imbalance.

## 3 Discussion

The present study has revealed considerable genetic heterogeneity at the *EDNRB2/mottling* locus, with evidence for at least three different alleles causing the mottling phenotype (Table 3), in addition to the Cys244Phe allele most likely causing tyrosinase‐independent recessive white (MO*W) in the Minohiki breed (Kinoshita et al. 2014). Given the distinct mutations in the *MO* gene between Japanese and French chicken breeds, indigenous chickens from other Asian, European, and African countries may harbor novel *MO* alleles awaiting discovery (Lalhlimpuia et al. 2021; Mahardhika et al. 2021; Chebo et al. 2023; Pavlova and Lukanov 2024; Begna et al. 2025; Touko et al. 2025). There are several other examples of allelic series at pigmentation loci in chickens such as *MC1R/Extension*, *SLC45A2/Silver*, and *PMEL/Dominant White* (reviewed by Andersson et al. 2020). These highly variable genes are all coding for transmembrane receptors, in which missense mutations may have quite variable consequences depending on their position in the protein.

We identified the missense mutation Ala228Thr in *EDNRB2* as a strong candidate causal mutation for recessive mottling in Mottled Houdan, Gournay, an Icelandic native mottled chicken, and Mottled Java (Table 3). This amino acid residue is located in the fourth transmembrane domain of EDNRB2 protein (https://www.uniprot.org/) and the corresponding codon shows a high degree of sequence conservation (PhyloP score = 6.26). The missense mutation showed complete association with mottling in two independent experimental populations. However, we also found evidence for *cis*‐regulatory mutation(s) affecting the expression of the *MO* allele in Mottled Houdan chicken, suggesting that the mottling phenotype in this breed may be caused by the combined effect of a mild defect in protein function and downregulation of gene expression. Ala228Thr is the only coding candidate mutation in IBD1, while the first 10 variants in Table S2 are candidate regulatory mutations for *EDNRB2*. The size of white spots varied considerably among birds homozygous for the Ala228Thr mutation in the INRAE line (Figure S5), which could be due to the segregation at another unknown locus affecting melanocyte migration/survival. However, considering our data and those previously published in chickens (Kinoshita et al. 2014), another possibility is that this line segregates for two different *EDNRB2* alleles, both carrying Ala228Thr, that differ as regards associated regulatory mutations affecting the degree of white spotting.

There is multiple evidence supporting the relationship between *EDNRB2* and plumage phenotypes in birds. Although the plumage pattern of mottling is unique in chickens, similar phenotypes to the down pattern of *MO* chicks (Figure 1d) can be found in other avian species, which are associated with reduced *EDNRB2* expression (Wu et al. 2016; Xi et al. 2020, 2021; Yang et al. 2022, 2024; Maclary et al. 2023). In mammals, *EDNRB* is the homolog to avian *EDNRB2* and they share similar functions (Braasch et al. 2009). Mutation and reduced expression of *EDNRB* are causing the piebald phenotype in mice, which is similar to the down pattern of *MO* chicks (Yamada et al. 2006). Lower *EDNRB* expression may delay melanoblast migration and stimulate early maturation of melanocytes in mice (Shin et al. 1999; Lee et al. 2003).

Somes Jr. (1980) proposed that the *MO* allele has two main effects on adult plumage patterns. First, to inhibit the pigmentation which causes the white tip and second as an eumelanin inducer to produce a V‐shaped eumelanin band after the white tip. The remainder of the feather vane has the background color without the effect of *MO*. The feather pattern is tricolored when the background color is not black. When black, the V‐shaped black band will not be obvious with only the white tip standing out. Therefore, the phenotypic effect of *MO* in mottling and tricolored chickens is the same (Somes Jr. 1980). If such an effect is accomplished by reduced *EDNRB2* expression in the lower bulge of the developing feather follicle, which maintains the undifferentiated melanocytes (Lin et al. 2013), one can expect delayed migration, similar to those from the neural crest, resulting in the absence of melanocytes on the tip of the feather. Also, early differentiation of melanocytes may be responsible for the V‐shaped black bands.

The interactions between *MO*, *Extension* (i.e., *MC1R*, Kerje et al. 2003), and *lavender* (i.e., melanophilin (*MLPH*), Vaez et al. 2008) were also studied by Somes Jr. (1980). In general, the shape and distribution of white tips in mottling pattern is affected by *Extension*, while the color of V‐shaped black bands in tricolor pattern can be diluted by *lavender* (Somes Jr. 1980). Today, it is understood that these genes regulate three relatively independent yet sequential processes: EDNRB2 (*MO*) is critical for the presence of mature melanocytes (Pla et al. 2005; Harris et al. 2008), MC1R (*E*) primarily determines whether eumelanosomes or pheomelanosomes are produced within melanocytes (Kerje et al. 2003; Schwochow et al. 2021; McNamara et al. 2021), and MLPH (*lavender*) affects the structure of these melanosomes (Vaez et al. 2008). Functional coding variations in these three genes have been identified in chicken. Moreover, genetic heterogeneities associated with the same phenotypes at both the *MO* and *E* loci have been reported (Dávila et al. 2014; Horecka et al. 2024), providing valuable material for further investigation into their complex interactions.

## 4 Conclusions

In summary, multiple pieces of evidence support that the missense mutation Ala228Thr possibly combined with reduced expression of *EDNRB2* is causing mottling in Mottled Houdan, Mottled Java, Gournay, and the mottled Icelandic chicken. We have shown that other mottled breeds such as tricolored Booted Bantam and Japanese Black Mottled Bantam must carry other causal mutations. Thus, there is considerable genetic heterogeneity at the *EDNRB2*/*mottling* locus in chickens and further work with more samples is required to reveal additional causal mutations and test if downregulation of *EDNRB2* expression is a common mechanism for the mottling phenotype across breeds.

## 5 Methods

### 5.1 Animals

A linkage mapping population was initiated from nine chickens purchased from Murray McMurray Hatchery (www.mcmurrayhatchery.com, Webster City, Iowa, USA). They consisted of three Mottled Houdan males and six Black Langshan females. Matings between 25 F_1_ females and three F_0_ Houdan males produced 221 backcross chickens.

Skin and feather tissues for RNA isolation were collected from two 6‐week‐old F_1_ chickens from the mating between Mottled Houdan and Black Silkie, which were purchased from Ideal Poultry (http://www.idealpoultry.com/, Cameron, Texas, USA) and kept according to Texas A&M University's Poultry Research Center Procedures. Blood samples from the same two individuals were also collected for DNA isolation. The tissue sample collections followed the guidelines of the Institutional Animal Care and Use Committee at Texas A&M University College of Veterinary Medicine and Biomedical Sciences.

Additionally, an experimental chicken line derived from the Houdan breed and kept segregating for mottling phenotype at INRAE PEAT Poultry Experimental Facility of Tours (Nouzilly, France, https://doi.org/10.15454/1.5572326250887292E12) was sampled to study within‐family segregation of the Ala228Thr genotype. DNA was obtained from 18 progeny of 3 sires (2 mottled and 1 non‐mottled) and 7 dams (3 mottled and 4 non‐mottled). Progeny included 8 mottled birds, 1 of them exhibiting very large white spots like in the “pied” phenotype, and 10 not showing the mottled phenotype (Table S4).

### 5.2 SNP Microarray and Pooling (SNP‐MaP) Analysis

Classification of mottling and black plumage patterns in the backcross population was made by the same individual at hatch and at 12 weeks of age. DNA samples were isolated from blood of each chicken at 4 weeks of age, and then combined into two pooled DNA samples based on the plumage patterns. The SNP‐MaP analysis (Docherty et al. 2007) was described in (Li et al. 2021), except that 60 K SNP Illumina iSelect chicken array providing information of 57 636 SNPs was applied for *MO* mapping, instead of 600 K chicken SNP genotyping array.

### 5.3 Whole Genome Sequencing

WGS data of the two DNA Pools (BioSample Accession: SAMN13810355 and SAMN13810356) representing the parental individuals in our mapping population were available from our previous study (Li et al. 2020). In addition, WGS data were provided by the IMAGE consortium (Horizon 2020 grant agreement n° 677 353) for four birds of two French breeds exhibiting a black mottled phenotype, Houdan (2 birds) and Gournay (2 birds). The data were analyzed together with publicly available WGS data from 207 individuals or pooled samples (Table S1). The latter included 13 samples from mottled or tricolored chickens and 194 samples from chickens lacking the mottling phenotype (black or wild‐type). All Illumina paired‐end FASTQ data were aligned to the red junglefowl genome assembly version GalGal6 using BWA (version: 0.7.12, Li 2013), sorted with SAMtools (version: 1.6, Li et al. 2009), and variants were called with GATK HaplotypeCaller 3.8 (Poplin et al. 2018). Quality control (QC) of these variants was carried out for the purposes of phasing and linkage disequilibrium (LD) analysis, and followed the standard recommendation by GATK. The QC consisted of “QD < 2.0 || FS > 60.0 || MQ < 40.0 || MQRankSum < −12.5 || ReadPosRankSum < −8.0” of filter‐expression option in GATK VariantFiltration for SNPs, and “QD < 2.0 || FS > 200.0 || ReadPosRankSum < −20.0” of filter‐expression option in GATK VariantFiltration for indels. Structural Variants (SV) were called with LUMPY (version: 0.2.13) (Layer et al. 2014).

### 5.4 Linkage Mapping

Two rounds of linkage mapping were conducted, which generally followed the procedures described previously (Li et al. 2021). A set of 17 SNPs was selected for the two rounds of linkage mapping. Genotyping of the 8 SNPs for the first round of linkage mapping was accomplished by KASP (Semagn et al. 2014), and results were validated by at least two amplification runs for each sample. The nine SNPs for the second round of linkage mapping were genotyped via standard PCR and Sanger sequencing (Table S5). Linkage mapping was carried out using CRIMAP (Green et al. 1990).

### 5.5 Identification of IBD Regions

We searched for the presence of IBD regions, expected to harbor causal mutations, using 18 WGS samples from mottled chickens (Table S1). Firstly, biallelic SNPs called by GATK after QC were phased by beagle (version: 5.1) using 24 WGS samples (18 mottling, and 6 reference samples, i.e., 3 black, and 3 red junglefowl samples). Secondly, a Python script distMat.py (https://github.com/simonhmartin/genomics_general) was applied to calculate the pair‐wise genetic distances among the 24 samples. Lastly, step‐wise genetic distance trees were built by a R script. The genetic distances (50 kb window in a 25 kb step manner or 10 kb window in a 5 kb step manner) between the pool of mottled Houdan and each of the other 8 non‐Japanese black mottled, 3 tricolored Booted Bantam, and 6 reference samples, were plotted across the genomic region. For other subsets of samples, the genetic distances were also selected and plotted. Genomic regions with high sequence identities (> 0.95) among a subset of mottling samples were considered as IBD regions.

### 5.6 Diagnostic Tests

Diagnostic tests to determine the strength of association between the candidate mutations and the mottling phenotypes were conducted using samples of 258 chickens representing 73 different populations and experimental lines. Five of the populations show the mottling phenotype while 68 are not mottled (Table S3). Two non‐synonymous mutations in *EDNRB2*, Ala228Thr and Arg332His, were genotyped using KASP assays. In addition, the Ala228Thr was also genotyped by pyrosequencing on the 18 birds from the INRAE segregating line.

### 5.7 Allelic Imbalance Test

Total RNA was extracted using the Quick‐RNA Miniprep Plus Kit (Zymo Research). First‐strand cDNA was synthesized using SuperScript IV VILO Master Mix (Invitrogen). Three PCR primers were designed to quantify the allelic imbalance of *EDNRB2* expression: genomic DNA (gDNA) was amplified using EDNRB2_AI_F1 (5′‐tatcgagcagtggcctcct‐3′) and EDNRB2_AI_R1 (5′‐tttctgttcagaggcaagca‐3′), while cDNA was amplified using EDNRB2_AI_F2 (5′‐cttggcacttggagaccttc‐3′) and the same reverse primer as used for gDNA. PCR products from four cDNA samples which came from two individuals, together with PCR products from gDNA of the same two individuals, were Sanger sequenced using the same reverse primer. The peak height for the Ala228Thr mutation was quantified using the PeakPicker 2 software (Ge et al. 2005). Allelic imbalance was estimated as the ratios of the peak height of the *N* allele over the *MO* allele in the cDNA and in the gDNA.

### 5.8 Identification of Candidate Mutations for Tricolored Samples

In order to narrow down the target region for the largest IBD region (at 9.5–10.8 Mb on chromosome 4), LD analysis by LDBlockShow (Dong et al. 2021) was carried out using the SNPs called by GATK with QC and excluding the ones with a missing rate larger than 0.25 and minor allele frequency below 0.01 in the three tricolored Booted Bantam samples plus the 195 black or WT samples (Table S1) (2476 SNPs for LD analysis). Moreover, allele frequency difference (dAF) for each of these SNPs was calculated comparing the three tricolored Booted Bantam samples and the 195 black or WT samples. Within all the target regions, variants called by GATK (without QC) and LUMPY were screened for complete association in the corresponding subset of samples (fixed in mottling samples while none of the 195 black or WT samples were homozygous), which were defined as candidate mutations for *MO*. The 77 vertebrates basewise PhyloP conservation scores (https://hgdownload.soe.ucsc.edu/goldenPath/galGal6/phastCons77way/) were utilized to predict the functional significance of candidate mutations.

## Author Contributions

**Chiara Bortoluzzi:** validation, writing – review and editing, investigation, resources. **David Gourichon:** resources. **Jingyi Li:** data curation, investigation, formal analysis, writing – original draft, writing – review and editing. **McKaela Hodge:** investigation, validation, formal analysis. **Leif Andersson:** conceptualization, writing – review and editing, supervision, funding acquisition, project administration. **Brian Davis:** supervision, software. **Michèle Tixier‐Boichard:** writing – review and editing, project administration, funding acquisition, validation. **Paul Siegel:** resources. **Bertrand Bed'hom:** methodology, resources. **Yifan Guo:** formal analysis, visualization. **Benjamin Dorshorst:** conceptualization, resources.

## Funding

This work was supported by Knut and Alice Wallenberg Foundation (KAW 2023.0160 to LA), Vetenskapsrådet (VR 2017‐02907 to LA). WGS sequences of the Houdan and the Gournay breed were funded by the Horizon 2020 program (grant # 677353 to MT‐B).

## Ethics Statement

Procedures for establishing the mapping population were approved by Institutional Animal Care and Use Committee at Virginia Tech. The animal experiments at INRA were carried out at the PEAT experimental unit under license number C37‐175‐1 for animal experimentation, in compliance with European Union legislation, and were approved by the local ethics committee for animal experimentation (Val de Loire) and by the French Ministries of Higher Education and Scientific Research, and Agriculture and Fisheries (n°2873–2 015 112 512 076 871), complying with the ARRIVE guidelines.

## Conflicts of Interest

The authors declare no conflicts of interest.

## Supporting information


**Figure S1:** Search of candidate region for *MO* in Japanese Black Mottled. Plots of pair‐wise genetic distances, based on variable sites only. The orange line indicates the contrast among individual sample of Japanese Black Mottled, grey lines indicate the contrast with other mottled samples (3 Houdan, 2 Gournay, 3 Java, 1 Icelandic native chicken, 3 tricolored Booted Bantam, 3 Orloff Red Spangled, and 1 Aseel Red Mottled), black lines indicate the contrast with non‐mottled samples (2 Black Java, 1 Black Leghorn, and 3 red junglefowl). The X‐axis is the genomic position on chromosome 4 in Mb. The previously reported candidate mutation for Japanese Black Mottled chickens is indicated in red.
**Figure S2:** Search of candidate region for *MO* in two Orloff Red Spangled samples. Plots of pair‐wise genetic distances, based on variable sites only. The blue line indicates the contrast among individual samples of Orloff Red Spangled, grey lines indicate the contrast with other mottled samples (3 Houdan, 2 Gournay, 3 Java, 1 Icelandic native chicken, 3 tricolored Booted Bantam, 2 Japanese Black Mottled, 1 Orloff Red Spangled, and 1 Aseel Red Mottled), black lines indicate the contrast with non‐mottled samples (2 Black Java, 1 Black Leghorn, and 3 red junglefowl). The X‐axis is the genomic position on chromosome 4 in Mb. The linkage mapping region based on the Mottled Houdan mapping population is indicated in red.
**Figure S3:** Search of candidate region for *MO* in one Orloff Red Spangled sample. Plots of pair‐wise genetic distances, based on variable sites only, between the individual sample of Orloff Red Spangled and other mottled or non‐mottled samples. Grey lines indicate the contrast with other mottled samples (3 Houdan, 2 Gournay, 3 Java, 1 Icelandic native chicken, 3 tricolored Booted Bantam, 2 Japanese Black Mottled, 2 Orloff Red Spangled, and 1 Aseel Red Mottled), black lines indicate the contrast with non‐mottled samples (2 Black Java, 1 Black Leghorn, and 3 red junglefowl). The X‐axis is the genomic position on chromosome 4 in Mb. The linkage mapping region based on the Mottled Houdan mapping population is indicated in red.
**Figure S4:** Search of candidate region for *MO* in Aseel Red Mottled. Plots of pair‐wise genetic distances, based on variable sites only. Grey lines indicate the contrast with other mottled samples (3 Houdan, 2 Gournay, 3 Java, 1 Icelandic native chicken, 3 tricolored Booted Bantam, 2 Japanese Black Mottled, and 3 Orloff Red Spangled), black lines indicate the contrast with non‐mottled samples (2 Black Java, 1 Black Leghorn, and 3 red junglefowl). The X‐axis is the genomic position on chromosome 4 in Mb. The linkage mapping region based on the Mottled Houdan mapping population is indicated in red.
**Figure S5:** Pictures of four progeny from the INRAE segregating family; birds are identified by their ID number given in Table S4. The genotype for the Ala228Thr mutation is shown for each bird. Bird #54 exhibited a much larger surface of white feathers than the other two birds homozygous for Ala228Thr.
**Table S1:** Whole genome sequence data used in this study.
**Table S2:** Summary of all candidate mutations for mottling in different breeds.
**Table S3:** Genotype data for the *EDNRB2* missense mutation Ala228Thr among 73 chicken populations.
**Table S4:** Pedigree and phenotype of the 18 chickens from the INRAE segregating line.
**Table S5:** PCR primers used for the second round of linkage mapping of the *MO* gene in Mottled Houdan chicken.

## Data Availability

Whole genome sequencing data of 2 Mottled Houdan, 2 Gournay are available on European Nucleotide Archive project PRJEB105336 (https://www.ebi.ac.uk/ena/browser/view/PRJEB105336) and one Icelandic mottled chicken is also available on European Nucleotide Archive project PRJNA552030 (https://www.ebi.ac.uk/ena/browser/view/PRJNA552030).
