# Supplementary material for: The Mottling Phenotype in Chickens Shows Genetic Heterogeneity and Is Caused by Mutations at the EDNRB2 Locus
Source: Anim Genet. 2026 Jul 17;57(4):e70168. doi: 10.1002/age.70168 (PMC13378286; doi:10.1002/age.70168)
Supplement: Supplementary file 1 — Figure S1: Search of candidate region for MO in Japanese Black Mottled. Plots of pair‐wise genetic distances, based on variable sites only. The orange line indicates the contrast among individual sample of Japanese Black Mottled, grey lines indicate the contrast with other mottled samples (3 Houdan, 2 Gournay, 3 Java, 1 Icelandic native chicken, 3 tricolored Booted Bantam, 3 Orloff Red Spangled, and 1 Aseel Red Mottled), black lines indicate the contrast with non‐mottled samples (2 Black Java, 1 Black Leghorn, and 3 red junglefowl). The X‐axis is the genomic position on chromosome 4 in Mb. The previously reported candidate mutation for Japanese Black Mottled chickens is indicated in red. Figure S2: Search of candidate region for MO in two Orloff Red Spangled samples. Plots of pair‐wise genetic distances, based on variable sites only. The blue line indicates the contrast among individual samples of Orloff Red Spangled, grey lines indicate the contrast with other mottled samples (3 Houdan, 2 Gournay, 3 Java, 1 Icelandic native chicken, 3 tricolored Booted Bantam, 2 Japanese Black Mottled, 1 Orloff Red Spangled, and 1 Aseel Red Mottled), black lines indicate the contrast with non‐mottled samples (2 Black Java, 1 Black Leghorn, and 3 red junglefowl). The X‐axis is the genomic position on chromosome 4 in Mb. The linkage mapping region based on the Mottled Houdan mapping population is indicated in red. Figure S3: Search of candidate region for MO in one Orloff Red Spangled sample. Plots of pair‐wise genetic distances, based on variable sites only, between the individual sample of Orloff Red Spangled and other mottled or non‐mottled samples. Grey lines indicate the contrast with other mottled samples (3 Houdan, 2 Gournay, 3 Java, 1 Icelandic native chicken, 3 tricolored Booted Bantam, 2 Japanese Black Mottled, 2 Orloff Red Spangled, and 1 Aseel Red Mottled), black lines indicate the contrast with non‐mottled samples (2 Black Java, 1 Black Leghorn, and 3 red junglefowl). Th [file AGE-57-0-s001.zip › age70168-sup-0006-FigureS1-S5-TableS1-S5@Mo_Supporting information.docx]

**Supporting information**

**
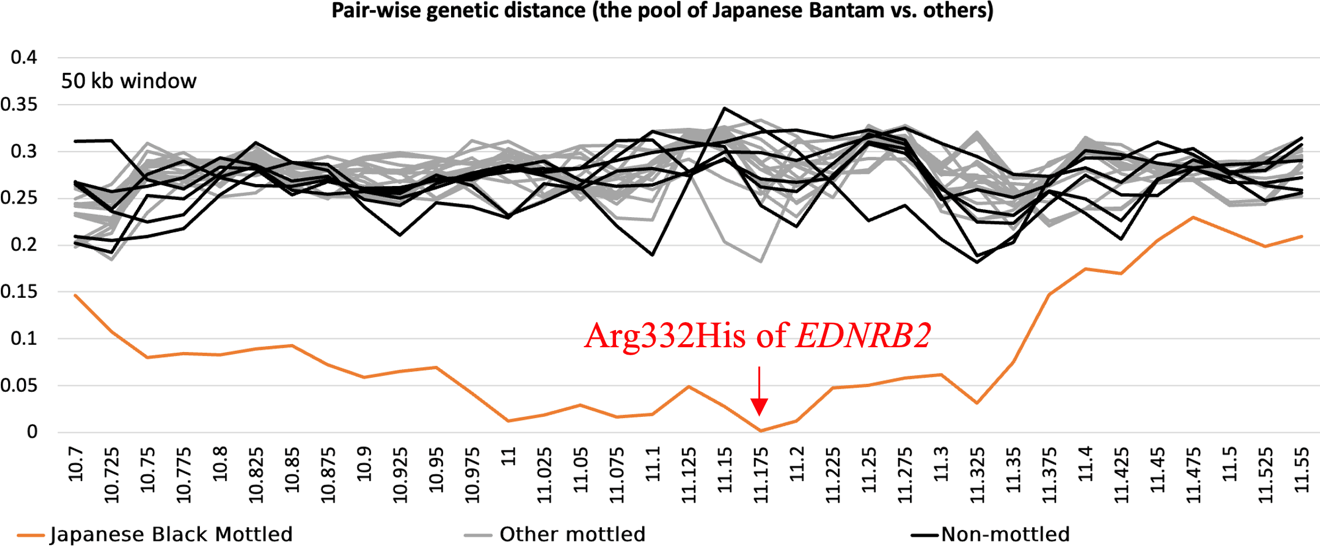
**

**Figure S1** Search of candidate region for *MO* in Japanese Black Mottled. Plots of pair-wise genetic distances, based on variable sites only. The orange line indicates the contrast among individual sample of Japanese Black Mottled, grey lines indicate the contrast with other mottled samples (3 Houdan, 2 Gournay, 3 Java, 1 Icelandic native chicken, 3 tricolored Booted Bantam, 3 Orloff Red Spangled, and 1 Aseel Red Mottled), black lines indicate the contrast with non-mottled samples (2 Black Java, 1 Black Leghorn, and 3 red junglefowl). The X-axis is the genomic position on chromosome 4 in Mb. The previously reported candidate mutation for Japanese Black Mottled chickens is indicated in red.


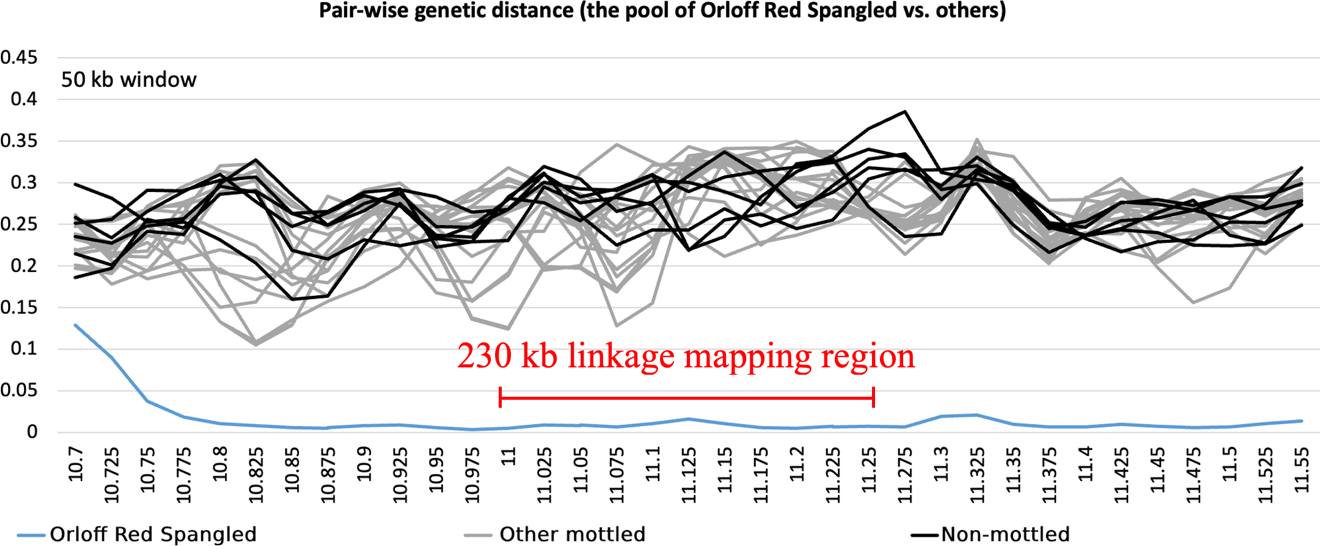


**Figure S2** Search of candidate region for *MO* in two Orloff Red Spangled samples. Plots of pair-wise genetic distances, based on variable sites only. The blue line indicates the contrast among individual samples of Orloff Red Spangled, grey lines indicate the contrast with other mottled samples (3 Houdan, 2 Gournay, 3 Java, 1 Icelandic native chicken, 3 tricolored Booted Bantam, 2 Japanese Black Mottled, 1 Orloff Red Spangled, and 1 Aseel Red Mottled), black lines indicate the contrast with non-mottled samples (2 Black Java, 1 Black Leghorn, and 3 red junglefowl). The X-axis is the genomic position on chromosome 4 in Mb. The linkage mapping region based on the Mottled Houdan mapping population is indicated in red.

**
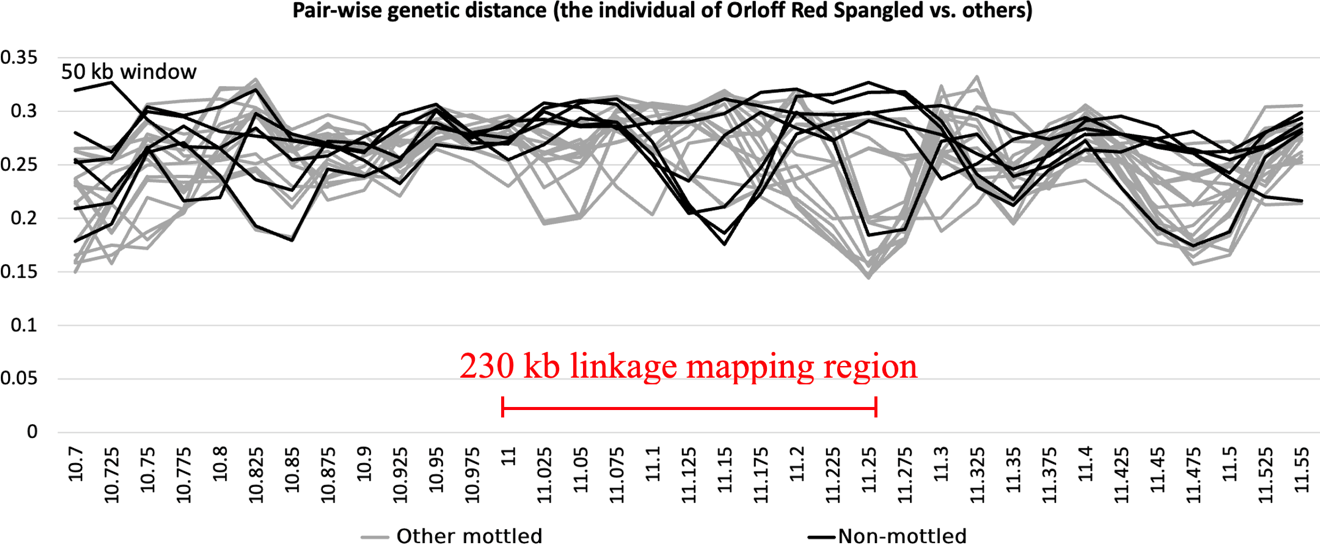
**

**Figure S3** Search of candidate region for *MO* in one Orloff Red Spangled sample. Plots of pair-wise genetic distances, based on variable sites only, between the individual sample of Orloff Red Spangled and other mottled or non-mottled samples. Grey lines indicate the contrast with other mottled samples (3 Houdan, 2 Gournay, 3 Java, 1 Icelandic native chicken, 3 tricolored Booted Bantam, 2 Japanese Black Mottled, 2 Orloff Red Spangled, and 1 Aseel Red Mottled), black lines indicate the contrast with non-mottled samples (2 Black Java, 1 Black Leghorn, and 3 red junglefowl). The X-axis is the genomic position on chromosome 4 in Mb. The linkage mapping region based on the Mottled Houdan mapping population is indicated in red.

**
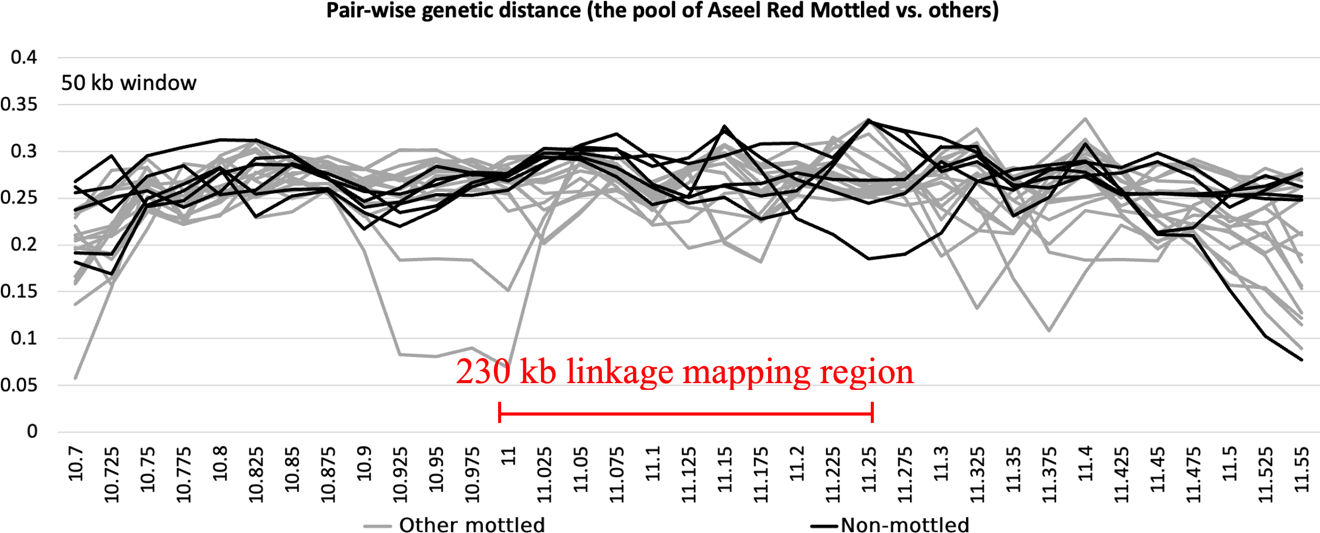
**

**Figure S4** Search of candidate region for *MO* in Aseel Red Mottled. Plots of pair-wise genetic distances, based on variable sites only. Grey lines indicate the contrast with other mottled samples (3 Houdan, 2 Gournay, 3 Java, 1 Icelandic native chicken, 3 tricolored Booted Bantam, 2 Japanese Black Mottled, and 3 Orloff Red Spangled), black lines indicate the contrast with non-mottled samples (2 Black Java, 1 Black Leghorn, and 3 red junglefowl). The X-axis is the genomic position on chromosome 4 in Mb. The linkage mapping region based on the Mottled Houdan mapping population is indicated in red.

**Figure S5** Pictures of four progeny from the INRAE segregating family; birds are identified by their ID number given in Table S4. The genotype for the Ala228Thr mutation is shown for each bird. Bird #54 exhibited a much larger surface of white feathers than the other two birds homozygous for Ala228Thr.

**Table S1. Whole genome sequence data used in this study**

| Breed | Variety | Library Type | BioSample Accession |
| --- | --- | --- | --- |
| Araucana | Black | Individual | SAMN11121887 |
| Araucana | Black | Pool | SAMEA104432183 |
| Aseel^1^ | Red Mottled^3^ | Pool | SAMEA104432184 |
| Booted Bantam |  | Individual | SAMEA6529991 |
| Booted Bantam^1^ | Mille Fleur^3^ | Individual | SAMEA6529990 |
| Booted Bantam^1^ | Mille Fleur^3^ | Pool | SAMEA104432193 |
| Booted Bantam^1^ | Red Porcelain^3^ | Individual | SAMEA5930879 |
| Castilian | Black | Individual | SAMEA6529931 |
| Castilian | Black | Pool | SAMEA104432198 |
| Cochin | Black | Individual | SAMN12236815 |
| Cochin | Black | Individual | SAMN12236812 |
| Cochin | Black | Individual | SAMN12236816 |
| Cochin | Black | Individual | SAMN12236813 |
| Cochin | Black | Individual | SAMN12236814 |
| Cochin | Black | Individual | SAMN12236819 |
| Cochin | Black | Individual | SAMN12236820 |
| Cochin | Black | Individual | SAMN12236817 |
| Cochin | Black | Individual | SAMN12236818 |
| Cochin | Black | Individual | SAMN12236821 |
| Cochin | Black | Pool | SAMEA104432189 |
| Creeper | Black | Pool | SAMEA104432200 |
| Gournay^1, 2^ | Black Mottled | Individual | SAMEA119581397 |
| Gournay^1, 2^ | Black Mottled | Individual | SAMEA119581407 |
| Houdan^1, 2^ | Black Mottled | Individual | SAMEA119581405 |
| Houdan^1, 2^ | Black Mottled | Individual | SAMEA119581406 |
| Houdan^1^ | Black Mottled | Pool | SAMN13810356 |
| Icelandic native^1, 2^ | Black Mottled | Individual | SAMN25706942 |
| Japanese Bantam^1^ | Black Mottled | Individual | SAMEA6529984 |
| Japanese Bantam^1^ | Black Mottled | Pool | SAMEA104432190 |
| Java | Auburn | Individual | SAMD00077882 |
| Java | Auburn | Individual | SAMD00077883 |
| Java | Auburn | Individual | SAMD00077890 |
| Java | Black | Individual | SAMD00077887 |
| Java | Black | Individual | SAMD00077888 |
| Java^1^ | Black Mottled | Individual | SAMD00077891 |
| Java^1^ | Black Mottled | Individual | SAMD00077884 |
| Java^1^ | Black Mottled | Individual | SAMD00077886 |
| Kedu Hitam |  | Individual | SAMD00077872 |
| Kedu Hitam |  | Individual | SAMD00077881 |
| Kedu Hitam |  | Individual | SAMD00077873 |
| Kedu Hitam |  | Individual | SAMD00077874 |
| Kedu Hitam |  | Individual | SAMD00077875 |
| Kedu Hitam |  | Individual | SAMD00077876 |
| Kedu Hitam |  | Individual | SAMD00077877 |
| Kedu Hitam |  | Individual | SAMD00077878 |
| Kedu Hitam |  | Individual | SAMD00077879 |
| Kedu Hitam |  | Individual | SAMD00077880 |
| Langshan | Black | Individual | SAMN12236872 |
| Langshan | Black | Individual | SAMN12236873 |
| Langshan | Black | Individual | SAMN12236874 |
| Langshan | Black | Individual | SAMN12236875 |
| Langshan | Black | Individual | SAMN12236876 |
| Langshan | Black | Individual | SAMN12236871 |
| Langshan | Black | Individual | SAMN12236868 |
| Langshan | Black | Individual | SAMN12236867 |
| Langshan | Black | Individual | SAMN12236870 |
| Langshan | Black | Individual | SAMN12236869 |
| Langshan | Black | Individual | SAMN12236866 |
| Langshan | Black | Individual | SAMN12236865 |
| Langshan | Black | Individual | SAMN12236877 |
| Langshan | Black | Individual | SAMN10471705 |
| Langshan | Black | Individual | SAMN10471706 |
| Langshan | Black | Individual | SAMN10471707 |
| Langshan | Black | Individual | SAMN10471708 |
| Langshan | Black | Individual | SAMN10471709 |
| Langshan | Black | Individual | SAMN10471710 |
| Langshan | Black | Individual | SAMN10471711 |
| Langshan | Black | Individual | SAMN10471712 |
| Langshan | Black | Pool | SAMN13810355 |
| Leghorn | Black | Individual | SAMEA6529930 |
| Minorca | Black | Individual | SAMN12236822 |
| Minorca | Black | Individual | SAMN12236827 |
| Minorca | Black | Individual | SAMN12236830 |
| Minorca | Black | Individual | SAMN12236829 |
| Minorca | Black | Individual | SAMN12236832 |
| Minorca | Black | Individual | SAMN12236831 |
| Minorca | Black | Individual | SAMN12236833 |
| Minorca | Black | Individual | SAMN12236834 |
| Minorca | Black | Individual | SAMN12236835 |
| Minorca | Black | Individual | SAMN12236824 |
| Minorca | Black | Individual | SAMN12236823 |
| Minorca | Black | Individual | SAMN12236826 |
| Minorca | Black | Individual | SAMN12236825 |
| Minorca | Black | Individual | SAMN12236828 |
| Orloff^1^ | Red Spangled^3^ | Individual | SAMEA6529962 |
| Orloff^1^ | Red Spangled^3^ | Individual | SAMN25706946 |
| Orloff^1^ | Red Spangled^3^ | Pool | SAMEA104432206 |
| Polish | White Crested Black | Individual | SAMN03177328 |
| Polish | White Crested Black | Individual | SAMEA6529996 |
| Red junglefowl | Burmese | Pool | SAMEA104432195 |
| Red junglefowl | India | Individual | SAMEA5160225 |
| Red junglefowl | India | Individual | SAMEA5160234 |
| Red junglefowl | India | Individual | SAMEA5160226 |
| Red junglefowl | India | Individual | SAMEA5160227 |
| Red junglefowl | India | Individual | SAMEA5160228 |
| Red junglefowl | India | Individual | SAMEA5160229 |
| Red junglefowl | India | Individual | SAMEA5160230 |
| Red junglefowl | India | Individual | SAMEA5160231 |
| Red junglefowl | India | Individual | SAMEA5160232 |
| Red junglefowl | India | Individual | SAMEA5160233 |
| Red junglefowl | Indochinese | Pool | SAMEA104432194 |
| Red junglefowl | Java | Individual | SAMD00077852 |
| Red junglefowl | Java | Individual | SAMD00077853 |
| Red junglefowl | Java | Individual | SAMD00077854 |
| Red junglefowl | Sumatra | Individual | SAMD00077855 |
| Red junglefowl | Sumatra | Individual | SAMD00077856 |
| Red junglefowl | Thailand | Individual | SAMEA5160200 |
| Red junglefowl | Thailand | Individual | SAMEA5160209 |
| Red junglefowl | Thailand | Individual | SAMEA5160210 |
| Red junglefowl | Thailand | Individual | SAMEA5160211 |
| Red junglefowl | Thailand | Individual | SAMEA5160212 |
| Red junglefowl | Thailand | Individual | SAMEA5160213 |
| Red junglefowl | Thailand | Individual | SAMEA5160214 |
| Red junglefowl | Thailand | Individual | SAMEA5160215 |
| Red junglefowl | Thailand | Individual | SAMEA5160216 |
| Red junglefowl | Thailand | Individual | SAMEA5160217 |
| Red junglefowl | Thailand | Individual | SAMEA5160218 |
| Red junglefowl | Thailand | Individual | SAMEA5160201 |
| Red junglefowl | Thailand | Individual | SAMEA5160219 |
| Red junglefowl | Thailand | Individual | SAMEA5160220 |
| Red junglefowl | Thailand | Individual | SAMEA5160221 |
| Red junglefowl | Thailand | Individual | SAMEA5160222 |
| Red junglefowl | Thailand | Individual | SAMEA5160223 |
| Red junglefowl | Thailand | Individual | SAMEA5160224 |
| Red junglefowl | Thailand | Individual | SAMEA5160202 |
| Red junglefowl | Thailand | Individual | SAMEA5160203 |
| Red junglefowl | Thailand | Individual | SAMEA5160204 |
| Red junglefowl | Thailand | Individual | SAMEA5160205 |
| Red junglefowl | Thailand | Individual | SAMEA5160206 |
| Red junglefowl | Thailand | Individual | SAMEA5160207 |
| Red junglefowl | Thailand | Individual | SAMEA5160208 |
| Red Junglefowl |  | Individual | SAMN14814745 |
| Red Junglefowl |  | Individual | SAMN14814746 |
| Red junglefowl |  | Individual | SAMN02486161 |
| Red junglefowl |  | Individual | SAMEA104717675 |
| Red junglefowl |  | Individual | SAMEA104285111 |
| Red junglefowl |  | Individual | SAMN14814812 |
| Red junglefowl |  | Individual | SAMN14814811 |
| Red junglefowl |  | Individual | SAMN14814810 |
| Red junglefowl |  | Individual | SAMN14814809 |
| Red junglefowl |  | Individual | SAMN14814808 |
| Red junglefowl |  | Individual | SAMN14814807 |
| Red junglefowl |  | Individual | SAMN14814806 |
| Red junglefowl |  | Individual | SAMN14814805 |
| Red Junglefowl |  | Individual | SAMN02333832 |
| Red junglefowl |  | Individual | SAMN14814804 |
| Red junglefowl |  | Individual | SAMN14814802 |
| Red junglefowl |  | Individual | SAMN14814801 |
| Red junglefowl |  | Individual | SAMN14814799 |
| Red junglefowl |  | Individual | SAMN14814798 |
| Red junglefowl |  | Individual | SAMN14814797 |
| Red junglefowl |  | Individual | SAMN14814796 |
| Red junglefowl |  | Individual | SAMN14814795 |
| Red junglefowl |  | Individual | SAMN14814794 |
| Red junglefowl |  | Individual | SAMN14814793 |
| Red Junglefowl |  | Individual | SAMN02333833 |
| Red junglefowl |  | Individual | SAMN14814792 |
| Red junglefowl |  | Individual | SAMN14814791 |
| Red junglefowl |  | Individual | SAMN14814790 |
| Red junglefowl |  | Individual | SAMN14814789 |
| Red junglefowl |  | Individual | SAMN14814788 |
| Red junglefowl |  | Individual | SAMN14814787 |
| Red junglefowl |  | Individual | SAMN14814786 |
| Red junglefowl |  | Individual | SAMN14814785 |
| Red junglefowl |  | Individual | SAMN14814784 |
| Red junglefowl |  | Individual | SAMN14814783 |
| Red Junglefowl |  | Individual | SAMN02712039 |
| Red junglefowl |  | Individual | SAMN14814782 |
| Red junglefowl |  | Individual | SAMN14814781 |
| Red junglefowl |  | Individual | SAMN14814780 |
| Red junglefowl |  | Individual | SAMN14814779 |
| Red junglefowl |  | Individual | SAMN14814778 |
| Red junglefowl |  | Individual | SAMN14814777 |
| Red junglefowl |  | Individual | SAMN14814776 |
| Red junglefowl |  | Individual | SAMN14814775 |
| Red junglefowl |  | Individual | SAMN14814774 |
| Red junglefowl |  | Individual | SAMN14814773 |
| Red Junglefowl |  | Individual | SAMN02712040 |
| Red junglefowl |  | Individual | SAMN14814772 |
| Red junglefowl |  | Individual | SAMN14814771 |
| Red junglefowl |  | Individual | SAMN14651083 |
| Red junglefowl |  | Individual | SAMN14651083 |
| Red junglefowl |  | Individual | SAMN14651083 |
| Red junglefowl |  | Individual | SAMN14651083 |
| Red junglefowl |  | Individual | SAMN14651083 |
| Red Junglefowl |  | Individual | SAMN02712041 |
| Red Junglefowl |  | Individual | SAMN02712042 |
| Red Junglefowl |  | Individual | SAMN02712043 |
| Red junglefowl |  | Individual | SAMN10471716 |
| Rosecomb Bantam | Black | Pool | SAMEA104432185 |
| Shamo | Black | Pool | SAMEA104432215 |
| Shouguang |  | Individual | SAMN02486162 |
| Sumatra | Black | Individual | SAMD00077862 |
| Sumatra | Black | Individual | SAMD00077871 |
| Sumatra | Black | Individual | SAMD00077863 |
| Sumatra | Black | Individual | SAMD00077864 |
| Sumatra | Black | Individual | SAMD00077865 |
| Sumatra | Black | Individual | SAMD00077866 |
| Sumatra | Black | Individual | SAMD00077867 |
| Sumatra | Black | Individual | SAMD00077868 |
| Sumatra | Black | Individual | SAMD00077869 |
| Sumatra | Black | Individual | SAMD00077870 |
| Sumatra | Black | Pool | SAMEA104432212 |
| Sumatra |  | Individual | SAMD00077857 |
| Sumatra |  | Individual | SAMD00077858 |
| Sumatra |  | Individual | SAMD00077859 |
| Sumatra |  | Individual | SAMD00077860 |
| Sumatra |  | Individual | SAMD00077861 |
| Sumatra |  | Individual | SAMEA6529953 |
| Yeonsan Ogye | Black | Individual | SAMN07344370 |
| Yeonsan Ogye | Black | Individual | SAMN07344371 |
| Yeonsan Ogye | Black | Individual | SAMN07344372 |
| Yeonsan Ogye | Black | Individual | SAMN07713967 |

^1^ Chicken samples with mottling phenotype. Others have pigmented plumage while no mottling phenotype (i.e. solid black or wild-type).

^2^ Sequenced in this study.

^3^ Tricolored phenotype.

**Table S2.** Summary of all candidate mutations for mottling in different breeds

| Associated IBD region | **Position on Chr. 4 (bp, GalGal6)** | dbSNP | Variant | N of heterozygotes in the 195 non-mottling samples | Cons. Score | Shared chicken samples | Annotation |
| --- | --- | --- | --- | --- | --- | --- | --- |
| IBD 1 | 11,146,555 | rs317604693 | C>T | 3 | -0.64 | 6 European black mottled and 3 Mottled Java | Exon of the lncRNA ENSGALG00000048079 |
| IBD 1 | 11,149,998 | rs312933187 | T>A | 1 | 0.01 | 6 European black mottled and 3 Mottled Java | Intron of the lncRNA ENSGALG00000048079 |
| IBD 1 | 11,150,903 | rs3385785194 | A>G | 2 | 0.10 | 6 European black mottled and 3 Mottled Java | Intron of the lncRNA ENSGALG00000048079 |
| IBD 1 | 11,152,293 | rs312938266 | G>A | 2 | 0.78 | 6 European black mottled and 3 Mottled Java | Intron of the lncRNA ENSGALG00000048079 |
| IBD 1 | 11,152,384 | rs3385782580 | A>G | 6 | -0.49 | 6 European black mottled and 3 Mottled Java | Intron of the lncRNA ENSGALG00000048079 |
| IBD 1 | 11,152,540 | rs3385799303 | T>G | 5 | -0.37 | 6 European black mottled and 3 Mottled Java | Intron of the lncRNA ENSGALG00000048079 |
| IBD 1 | 11,152,802 | rs317818377 | A>G | 2 | 0.10 | 6 European black mottled and 3 Mottled Java | Intron of the lncRNA ENSGALG00000048079 |
| IBD 1 | 11,155,328 | Not in dbSNP | A>AATC | 3 | -0.62 | 6 European black mottled and 3 Mottled Java | Intergenic |
| IBD 1 | 11,156,089 | rs1060311521 | T>C | 6 | -0.10 | 6 European black mottled and 3 Mottled Java | Intergenic |
| IBD 1 | 11,158,327 | rs315709540 | A>T | 8 | -0.04 | 6 European black mottled, 3 Mottled Java, and 1 Red Porcelain Booted Bantam | Intergenic |
| IBD 1 | 11,164,253 | rs313943998 | G>A | 0 | 6.26 | 6 European black mottled and 3 Mottled Java | Ala228Thr of *EDNRB2* |
| IBD 3 | 11,492,626 | rs315646632 | C>T | 5 | -1.74 | 3 tricolored Booted Bantams, 1 Mottled Java | Intron of the lncRNA ENSGALG00000050381 |
| IBD 3 | 11,492,715 | rs316433172 | C>T | 6 | -0.82 | 3 tricolored Booted Bantams, 1 Mottled Java | Exon of the lncRNA ENSGALG00000050382 |
| IBD 3 | 11,493,059 | rs738712443 | G>C | 5 | -0.15 | 3 tricolored Booted Bantams, 1 Mottled Java | Intron of the lncRNA ENSGALG00000050381 |
| IBD 3 | 11,493,504 | rs315194649 | G>A | 11 | 1.69 | 3 tricolored Booted Bantams, 1 Mottled Java, and 2 Red Spangled Orloff | Intron of the lncRNA ENSGALG00000050381 |
| IBD 3 | 11,504,162 | rs734898966 | C>T | 5 | -0.95 | 3 tricolored Booted Bantams, 1 Mottled Java | Intergenic |
| IBD 5 | 9,561,094 | rs740365086 | G>A | 3 | -1.93 | 3 tricolored Booted Bantams | Intron of *CLCN5* |
| IBD 5 | 10,620,087 | Not in dbSNP | C>T | 1 | 0.06 | 3 tricolored Booted Bantams |  |
| IBD 5 | 10,661,202 | Not in dbSNP | G>A | 0 | -3.83 | 3 tricolored Booted Bantams | Intron of *GABRQ* |
| IBD 5 | 10,705,922 | Not in dbSNP | T>C | 0 | -1.31 | 3 tricolored Booted Bantams | Intron of *GABRQ* |
| IBD 5 | 10,730,655 | rs738553274 | G>A | 3 | 0.36 | 3 tricolored Booted Bantams |  |
| IBD 5 | 10,738,572 | rs313562335 | C>A | 1 | 0.18 | 3 tricolored Booted Bantams |  |
| IBD 5 | 10,739,554 | rs741706486 | C>T | 18 | 1.17 | 3 tricolored Booted Bantams, 1 Mottled Java |  |
| - | 11,166,001 | Not in dbSNP | G>A | 0 | 6.4 | 2 Japanese mottled breeds | Arg332His of *EDNRB2* |

**Table S3. Genotype data for the *EDNRB2* missense mutation Ala228Thr among 73 chicken populations.**

| **Breed** | **Plumage color** | **Source** | **Mottling allele** | **Ala228Thr Genotype** | | | **Total** |
| --- | --- | --- | --- | --- | --- | --- | --- |
|  |  |  |  | **G/G (Ala/Ala)** | **G/A (Ala/Thr)** | **A/A (Thr/Thr)** |  |
| Ameraucana | Black | Lucasville, OH, U.S. | *MO*N* | 2 |  |  | 2 |
| Ameraucana | Blue | Lucasville, OH, U.S. | *MO*N* | 2 |  |  | 2 |
| American Longtail | Wild-type | H & H Longtails | *MO*N* | 8 | 2 |  | 10 |
| Andalusian | Blue | Marshfield, WI, U.S. | *MO*N* | 1 |  |  | 1 |
| Australorp | Black | Marshfield, WI, U.S. | *MO*N* | 1 |  |  | 1 |
| Ayam Cemani | Black | Minnesota, U.S. | *MO*N* | 7 |  |  | 7 |
| B (Wild-type) | Wild-type | Arizona, U.S. | *MO*N* | 7 |  |  | 7 |
| Brahma | Buff | Macclesfield, NC, USA | *MO*N* | 2 |  |  | 2 |
| Brahma | Dark | Lucasville, OH, U.S. | *MO*N* | 1 |  |  | 1 |
| Brahma | Light | Raleigh, NC, USA | *MO*N* | 2 |  |  | 2 |
| Buttercup | Buttercup | Murray McMurray | *MO*N* | 10 |  |  | 9 |
| Buttercup | Buttercup | Lucasville, OH, U.S. | *MO*N* | 9 |  |  | 10 |
| C (Black) | Black | Arizona, U.S. | *MO*N* | 4 | 4 |  | 8 |
| Campine | Golden | Raleigh, NC, USA | *MO*N* | 2 |  |  | 2 |
| Chantecler | Partridge | Lucasville, OH, U.S. | *MO*N* | 1 |  |  | 1 |
| Cochin | Buff | Macclesfield, NC, USA | *MO*N* | 1 |  |  | 1 |
| Cochin | Golden Laced | Lucasville, OH, U.S. | *MO*N* | 1 |  |  | 1 |
| Cochin | Partridge | Murray McMurray | *MO*N* | 2 |  |  | 2 |
| Cornish | Dark | Murray McMurray | *MO*N* | 10 |  |  | 10 |
| D (Splash) | Splash | Arizona, U.S. | *MO*N* | 10 |  |  | 10 |
| Dorking | Silver Gray | Murray McMurray | *MO*N* | 6 |  |  | 6 |
| Faverolles | Salmon | Murray McMurray | *MO*N* | 6 |  |  | 6 |
| Frizzle | Red | Raleigh, NC, USA | *MO*N* | 1 |  |  | 1 |
| Hamburg | Golden Penciled | Murray McMurray | *MO*N* | 2 |  |  | 2 |
| Hamburg | Silver Spangled | Murray McMurray | *MO*N* | 10 |  |  | 10 |
| Hamburg | Silver Spangled | Macclesfield, NC, USA | *MO*N* | 2 |  |  | 2 |
| Junglefowl | Red | Richardson line | *MO*N* | 6 |  |  | 6 |
| Langshan | Black | Murray McMurray | *MO*N* | 10 |  |  | 2 |
| Langshan | Black | Lucasville, OH, U.S. | *MO*N* | 2 |  |  | 10 |
| Leghorn | Black tail, Red | Lucasville, OH, U.S. | *MO*N* | 2 |  |  | 2 |
| Leghorn | Single Comb Dark Brown | Raleigh, NC, USA | *MO*N* | 1 |  |  | 1 |
| Leghorn | Single Comb Light Brown | Raleigh, NC, USA | *MO*N* | 1 |  |  | 1 |
| Leghorn | Single Comb Light Brown | Lucasville, OH, U.S. | *MO*N* | 1 |  |  | 1 |
| Marans | Birchen | Greenfire Farms | *MO*N* | 3 |  |  | 3 |
| Marans | Birchen | Virginia Tech | *MO*N* | 5 |  |  | 5 |
| Marans | Wheaten | Lucasville, OH, U.S. | *MO*N* | 2 |  |  | 2 |
| Marans | Copper | Lucasville, OH, U.S. | *MO*N* | 2 |  |  | 2 |
| Orpington | Black | Marshfield, WI, U.S. | *MO*N* | 1 |  |  | 1 |
| Orpington | Buff | Macclesfield, NC, USA | *MO*N* | 1 |  |  | 2 |
| Orpington | Buff | Lucasville, OH, U.S. | *MO*N* | 2 |  |  | 1 |
| Phoenix | Silver | Marshfield, WI, U.S. | *MO*N* | 1 |  |  | 1 |
| Plymouth Rock | Barred | Raleigh, NC, USA | *MO*N* | 1 |  |  | 2 |
| Plymouth Rock | Barred | Virginia Tech | *MO*N* | 6 |  |  | 1 |
| Plymouth Rock | Barred | Raleigh, NC, USA | *MO*N* | 2 |  |  | 6 |
| Plymouth Rock | Partridge | Murray McMurray | *MO*N* | 10 |  |  | 10 |
| Plymouth Rock | Silver Penciled | Murray McMurray | *MO*N* | 2 |  |  | 2 |
| Polish | Buff Laced | Macclesfield, NC, USA | *MO*N* | 2 |  |  | 2 |
| Polish | White Crested Black | Raleigh, NC, USA | *MO*N* | 2 |  |  | 2 |
| Polish | White Crested Black | Marshfield, WI, U.S. | *MO*N* | 1 |  |  | 1 |
| Polish | White Crested Black | Lucasville, OH, U.S. | *MO*N* | 1 |  |  | 1 |
| Polish | White Crested Blue | Lucasville, OH, U.S. | *MO*N* | 1 |  |  | 1 |
| Rhode Island | Red | Macclesfield, NC, USA | *MO*N* | 2 |  |  | 1 |
| Rhode Island | Red | Lucasville, OH, U.S. | *MO*N* | 1 |  |  | 2 |
| Sebright | Golden | Macclesfield, NC, USA | *MO*N* | 2 |  |  | 1 |
| Sebright | Golden | Lucasville, OH, U.S. | *MO*N* | 1 |  |  | 2 |
| Sebright | Silver | Murray McMurray | *MO*N* | 10 |  |  | 10 |
| Sebright | Silver | Macclesfield, NC, USA | *MO*N* | 2 |  |  | 2 |
| Silkie | Black | Raleigh, NC, USA | *MO*N* | 5 |  |  | 5 |
| Spitzhauben | German | Raleigh, NC, USA | *MO*N* | 2 |  |  | 2 |
| Sumatra | Black | Lucasville, OH, U.S. | *MO*N* | 2 |  |  | 2 |
| Sumatra | Black | Raleigh, NC, USA | *MO*N* | 10 |  |  | 10 |
| Sumatra | Blue | Lucasville, OH, U.S. | *MO*N* | 2 |  |  | 2 |
| Sumatra | Blue | Marshfield, WI, U.S. | *MO*N* | 1 |  |  | 1 |
| Svarthöna | Black | Uppsala, Sweden | *MO*N* | 4 |  |  | 4 |
| Wyandotte | Chocolate | Greenfire Farms | *MO*N* | 5 |  |  | 5 |
| Wyandotte | Partridge | Murray McMurray | *MO*N* | 2 |  |  | 2 |
| Wyandotte | Silver Penciled | Murray McMurray | *MO*N* | 1 |  |  | 1 |
| Yokohama | Red Shoulder | Lucasville, OH, U.S. | *MO*N* | 1 |  |  | 1 |
| D'Uccle Belgian | Mille Fleur | Lucasville, OH, U.S. | *MO*MO* | 2 |  |  | 2 |
| Houdan | Mottled | Murray McMurray | *MO*MO* |  |  | 16 | 16 |
| Houdan | Mottled | Lucasville, OH, U.S. | *MO*MO* |  |  | 1 | 1 |
| Old English | Spangled | Lucasville, OH, U.S. | *MO*MO* | 1 |  |  | 1 |
| Sussex | Speckled | Macclesfield, NC, USA | *MO*MO* | 1 |  |  | 1 |
|  |  |  |  |  | Grand Total | | 258 |

**Table S4.** Pedigree and phenotype of the 18 chickens from the INRAE segregating line

| **sire ID** | **sire phenotype** | **dam ID** | **dam phenotype** | **progeny ID** | **progeny phenotype and genotype for Ala228Thr** | |
| --- | --- | --- | --- | --- | --- | --- |
| 5080 | non-mottled | 5146 | mottled | 1 | a few white spots | A/G |
| 5080 | non-mottled | 5146 | mottled | 3 | no white spots | A/G |
| 5080 | non-mottled | 5142 | non-mottled | 5 | faint white spots | A/G |
| 5080 | non-mottled | 5142 | non-mottled | 7 | mottled (regular white spots) | A/A |
| 5080 | non-mottled | 5209 | mottled | 13 | no white spots | A/G |
| 5080 | non-mottled | 5089 | mottled | 17 | no white spots | A/G |
| 5080 | non-mottled | 5089 | mottled | 21 | a few white spots^1^ | A/G |
| 5080 | non-mottled | 5188 | mottled | 27 | no white spots | A/G |
| 5080 | non-mottled | 5188 | mottled | 29 | no white spots | A/G |
| 5057 | mottled | 5071 | non-mottled | 36 | mottled (white spots + white belly) ^1^ | A/A |
| 5057 | mottled | 5071 | non-mottled | 40 | mottled (white spots + white belly + white areas on the back) ^1^ | A/A |
| 5057 | mottled | 5071 | non-mottled | 43 | no white spots | A/G |
| 5057 | mottled | 5071 | non-mottled | 45 | large white spots all over the body | A/A |
| 5152 | mottled | 8533 | non-mottled | 48 | mottled (white spots + white belly + white areas on the back) | A/A |
| 5152 | mottled | 8533 | non-mottled | 50 | mottled (white spots + white belly + white areas on the back) | A/A |
| 5152 | mottled | 8533 | non-mottled | 54 | mottled (white spots + white belly + white areas on the back) ^1^ | A/A |
| 5152 | mottled | 8533 | non-mottled | 56 | mottled (white spots + white belly) | A/A |
| 5152 | mottled | 8533 | non-mottled | 58 | a few white spots | A/G |

^1^ A picture is provided in Figure S5.

**Table S5.** PCR primers used for the second round of linkage mapping of the *MO* gene in Mottled Houdan chicken

| **Target SNP** | **Position on Chr. 4 (bp, GalGal6)** | **Primer Name** | **Primer Sequence (5'-3')** | **Product**  **Size (bp)** |
| --- | --- | --- | --- | --- |
| rs313406325 | 10,856,646 | mo_085F1 | ATGCTATGCTCCCACCCAGA | 395 |
|  |  | mo_085R1 | AGTGCAATGGCTTGGATGCT |  |
| rs736157261 | 11,007,003 | mo_100F1 | AAGCAGAGCACCTGCAAAGG | 638 |
|  |  | mo_100R1 | AGGGAGAGGCTGGTGGAAAC |  |
| rs16361338 | 11,122,775 | mo_112F1 | GAGGAAGAGACCGCCAACACT | 568 |
|  |  | mo_112R1 | ACATTCCCACCAAGCTCGATT |  |
| rs317986873 | 11,172,708 | mo_117F1 | TGCCAGCCTATCACCAACAGT | 966 |
|  |  | mo_117R1 | TTTCTCTCTCTCCCCACCACAG |  |
| rs314405946 | 11,198,832 | mo_120F1 | GCGTGGCTTTTGCTGATTG | 592 |
|  |  | mo_120R1 | ATCCCCAGGCATCTGCTGTA |  |
| rs313821755 | 11,241,378 | mo_124F1 | TTGGGCAGAGCTGACAGTGA | 500 |
|  |  | mo_124R1 | CTGGGAGCTGCTGTTTGAGG |  |
| rs16361521 | 11,275,557 | mo_128F1 | TGCTGCAATCCAACATCCTG | 657 |
|  |  | mo_128R1 | GCATCAGTGGGGAAAGCAAA |  |
| rs15494340 | 11,311,842 | mo_131F1 | CGCAGGAAAAGCCAACTCAC | 664 |
|  |  | mo_131R1 | GCCATCACAGGCAGAAATCC |  |
| rs313834381 | 11,461,125 | mo_146F1 | GGGTTCCCTTTCTCCACCAG | 910 |
|  |  | mo_146R1 | CTCACCTTCCTGAGCCATGC |  |
