# Supplementary figures and images for: The Mottling Phenotype in Chickens Shows Genetic Heterogeneity and Is Caused by Mutations at the EDNRB2 Locus
Source: Anim Genet. 2026 Jul 17;57(4):e70168. doi: 10.1002/age.70168 (PMC13378286; doi:10.1002/age.70168)

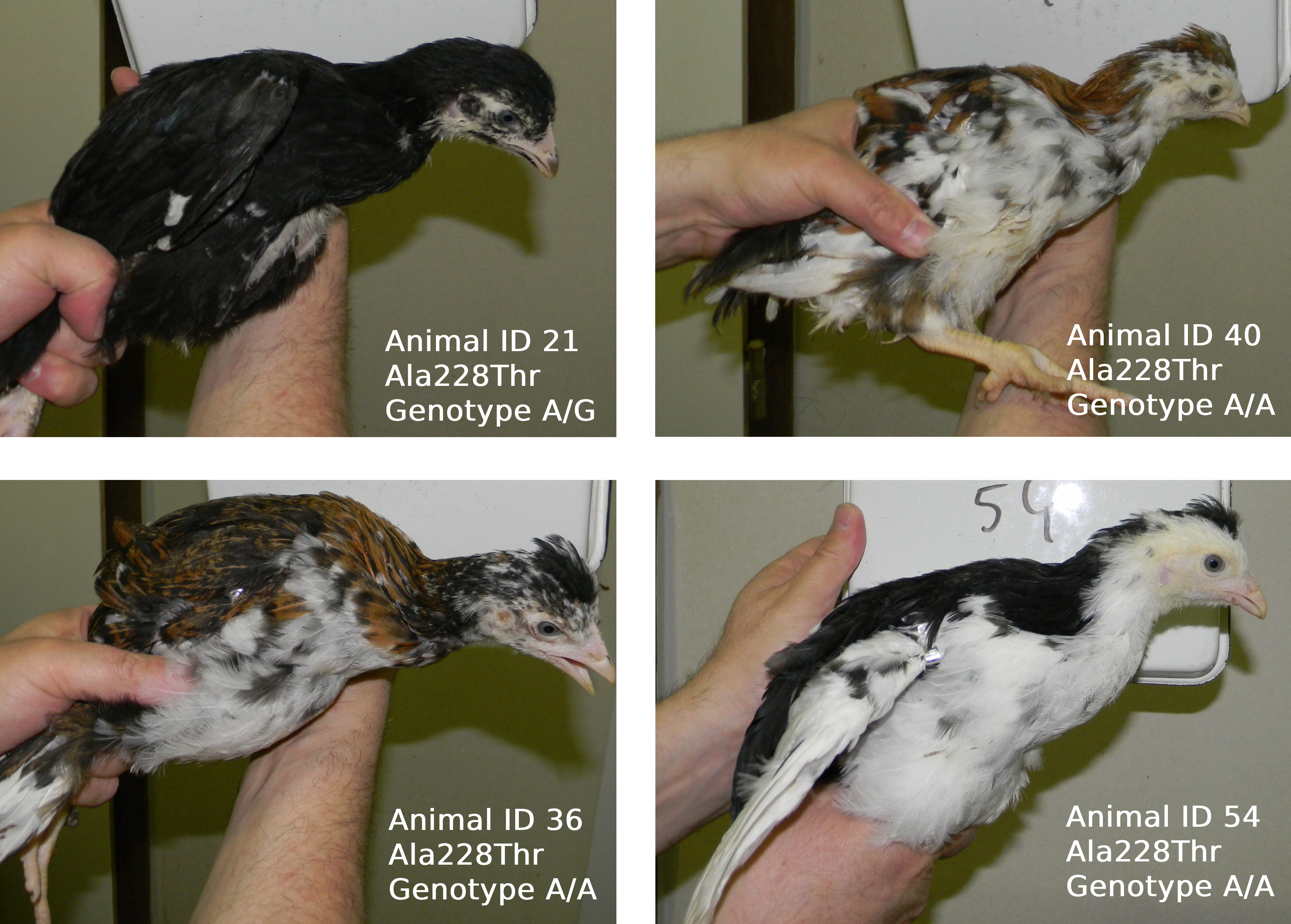

Supplement: Supplementary file 1 — Figure S1: Search of candidate region for MO in Japanese Black Mottled. Plots of pair‐wise genetic distances, based on variable sites only. The orange line indicates the contrast among individual sample of Japanese Black Mottled, grey lines indicate the contrast with other mottled samples (3 Houdan, 2 Gournay, 3 Java, 1 Icelandic native chicken, 3 tricolored Booted Bantam, 3 Orloff Red Spangled, and 1 Aseel Red Mottled), black lines indicate the contrast with non‐mottled samples (2 Black Java, 1 Black Leghorn, and 3 red junglefowl). The X‐axis is the genomic position on chromosome 4 in Mb. The previously reported candidate mutation for Japanese Black Mottled chickens is indicated in red. Figure S2: Search of candidate region for MO in two Orloff Red Spangled samples. Plots of pair‐wise genetic distances, based on variable sites only. The blue line indicates the contrast among individual samples of Orloff Red Spangled, grey lines indicate the contrast with other mottled samples (3 Houdan, 2 Gournay, 3 Java, 1 Icelandic native chicken, 3 tricolored Booted Bantam, 2 Japanese Black Mottled, 1 Orloff Red Spangled, and 1 Aseel Red Mottled), black lines indicate the contrast with non‐mottled samples (2 Black Java, 1 Black Leghorn, and 3 red junglefowl). The X‐axis is the genomic position on chromosome 4 in Mb. The linkage mapping region based on the Mottled Houdan mapping population is indicated in red. Figure S3: Search of candidate region for MO in one Orloff Red Spangled sample. Plots of pair‐wise genetic distances, based on variable sites only, between the individual sample of Orloff Red Spangled and other mottled or non‐mottled samples. Grey lines indicate the contrast with other mottled samples (3 Houdan, 2 Gournay, 3 Java, 1 Icelandic native chicken, 3 tricolored Booted Bantam, 2 Japanese Black Mottled, 2 Orloff Red Spangled, and 1 Aseel Red Mottled), black lines indicate the contrast with non‐mottled samples (2 Black Java, 1 Black Leghorn, and 3 red junglefowl). Th [file AGE-57-0-s001.zip › age70168-sup-0005-FigureS5@Figure S5.png]

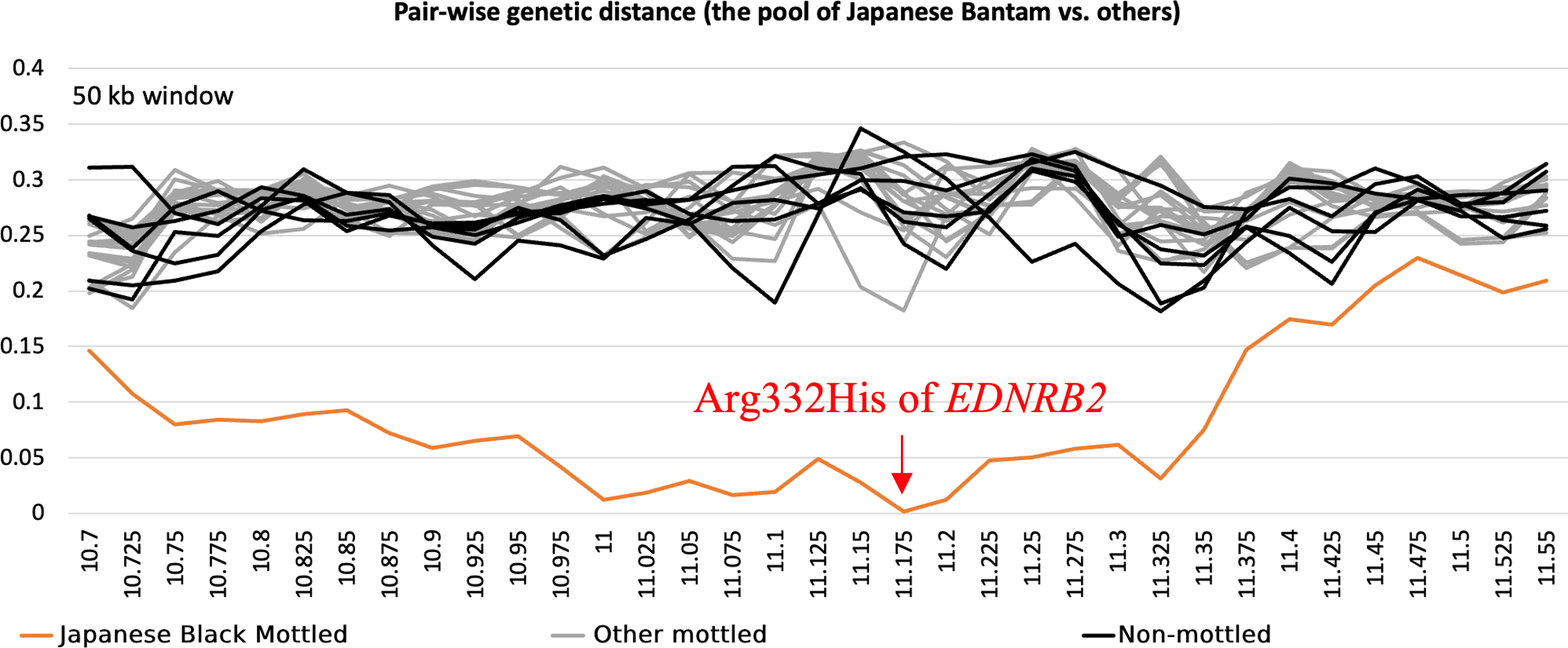

Supplement: Supplementary file 1 — Figure S1: Search of candidate region for MO in Japanese Black Mottled. Plots of pair‐wise genetic distances, based on variable sites only. The orange line indicates the contrast among individual sample of Japanese Black Mottled, grey lines indicate the contrast with other mottled samples (3 Houdan, 2 Gournay, 3 Java, 1 Icelandic native chicken, 3 tricolored Booted Bantam, 3 Orloff Red Spangled, and 1 Aseel Red Mottled), black lines indicate the contrast with non‐mottled samples (2 Black Java, 1 Black Leghorn, and 3 red junglefowl). The X‐axis is the genomic position on chromosome 4 in Mb. The previously reported candidate mutation for Japanese Black Mottled chickens is indicated in red. Figure S2: Search of candidate region for MO in two Orloff Red Spangled samples. Plots of pair‐wise genetic distances, based on variable sites only. The blue line indicates the contrast among individual samples of Orloff Red Spangled, grey lines indicate the contrast with other mottled samples (3 Houdan, 2 Gournay, 3 Java, 1 Icelandic native chicken, 3 tricolored Booted Bantam, 2 Japanese Black Mottled, 1 Orloff Red Spangled, and 1 Aseel Red Mottled), black lines indicate the contrast with non‐mottled samples (2 Black Java, 1 Black Leghorn, and 3 red junglefowl). The X‐axis is the genomic position on chromosome 4 in Mb. The linkage mapping region based on the Mottled Houdan mapping population is indicated in red. Figure S3: Search of candidate region for MO in one Orloff Red Spangled sample. Plots of pair‐wise genetic distances, based on variable sites only, between the individual sample of Orloff Red Spangled and other mottled or non‐mottled samples. Grey lines indicate the contrast with other mottled samples (3 Houdan, 2 Gournay, 3 Java, 1 Icelandic native chicken, 3 tricolored Booted Bantam, 2 Japanese Black Mottled, 2 Orloff Red Spangled, and 1 Aseel Red Mottled), black lines indicate the contrast with non‐mottled samples (2 Black Java, 1 Black Leghorn, and 3 red junglefowl). Th [file AGE-57-0-s001.zip › age70168-sup-0001-FigureS1@Figure S1.png]

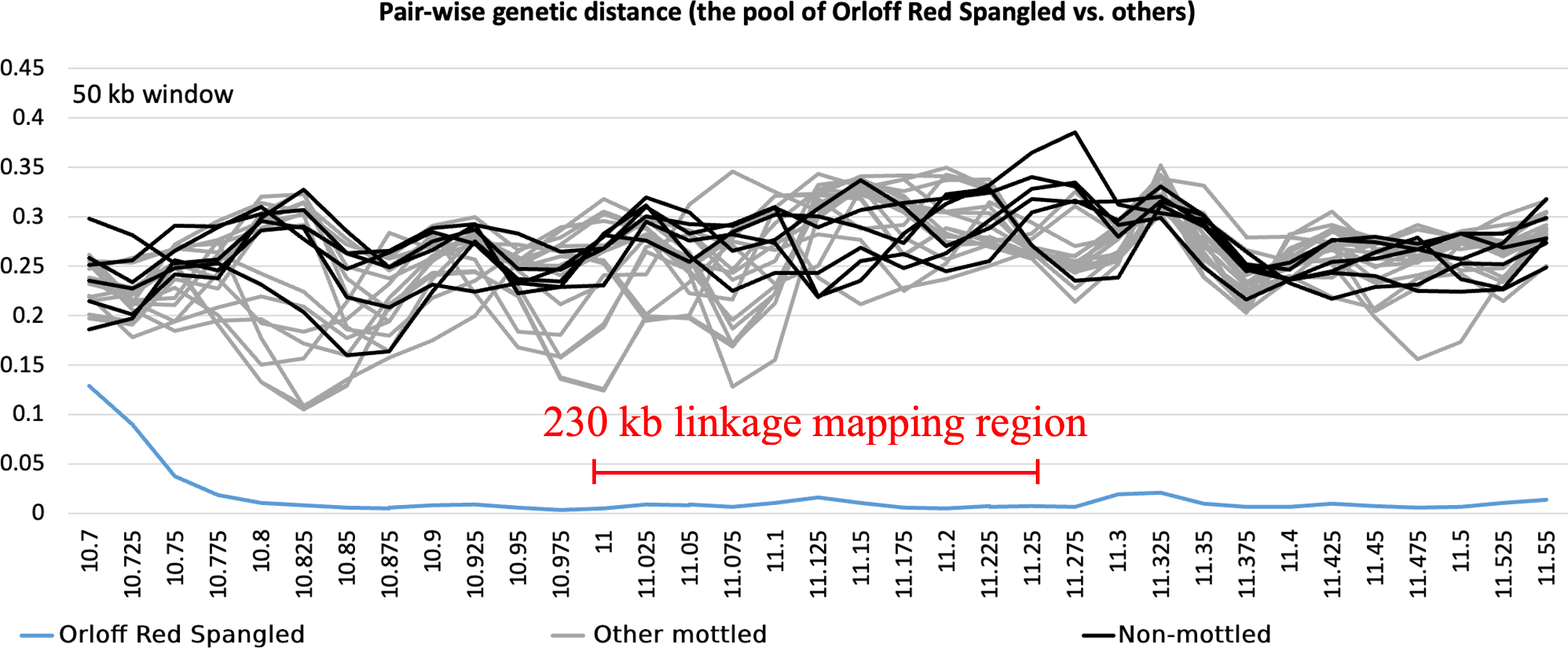

Supplement: Supplementary file 1 — Figure S1: Search of candidate region for MO in Japanese Black Mottled. Plots of pair‐wise genetic distances, based on variable sites only. The orange line indicates the contrast among individual sample of Japanese Black Mottled, grey lines indicate the contrast with other mottled samples (3 Houdan, 2 Gournay, 3 Java, 1 Icelandic native chicken, 3 tricolored Booted Bantam, 3 Orloff Red Spangled, and 1 Aseel Red Mottled), black lines indicate the contrast with non‐mottled samples (2 Black Java, 1 Black Leghorn, and 3 red junglefowl). The X‐axis is the genomic position on chromosome 4 in Mb. The previously reported candidate mutation for Japanese Black Mottled chickens is indicated in red. Figure S2: Search of candidate region for MO in two Orloff Red Spangled samples. Plots of pair‐wise genetic distances, based on variable sites only. The blue line indicates the contrast among individual samples of Orloff Red Spangled, grey lines indicate the contrast with other mottled samples (3 Houdan, 2 Gournay, 3 Java, 1 Icelandic native chicken, 3 tricolored Booted Bantam, 2 Japanese Black Mottled, 1 Orloff Red Spangled, and 1 Aseel Red Mottled), black lines indicate the contrast with non‐mottled samples (2 Black Java, 1 Black Leghorn, and 3 red junglefowl). The X‐axis is the genomic position on chromosome 4 in Mb. The linkage mapping region based on the Mottled Houdan mapping population is indicated in red. Figure S3: Search of candidate region for MO in one Orloff Red Spangled sample. Plots of pair‐wise genetic distances, based on variable sites only, between the individual sample of Orloff Red Spangled and other mottled or non‐mottled samples. Grey lines indicate the contrast with other mottled samples (3 Houdan, 2 Gournay, 3 Java, 1 Icelandic native chicken, 3 tricolored Booted Bantam, 2 Japanese Black Mottled, 2 Orloff Red Spangled, and 1 Aseel Red Mottled), black lines indicate the contrast with non‐mottled samples (2 Black Java, 1 Black Leghorn, and 3 red junglefowl). Th [file AGE-57-0-s001.zip › age70168-sup-0002-FigureS2@Figure S2.png]

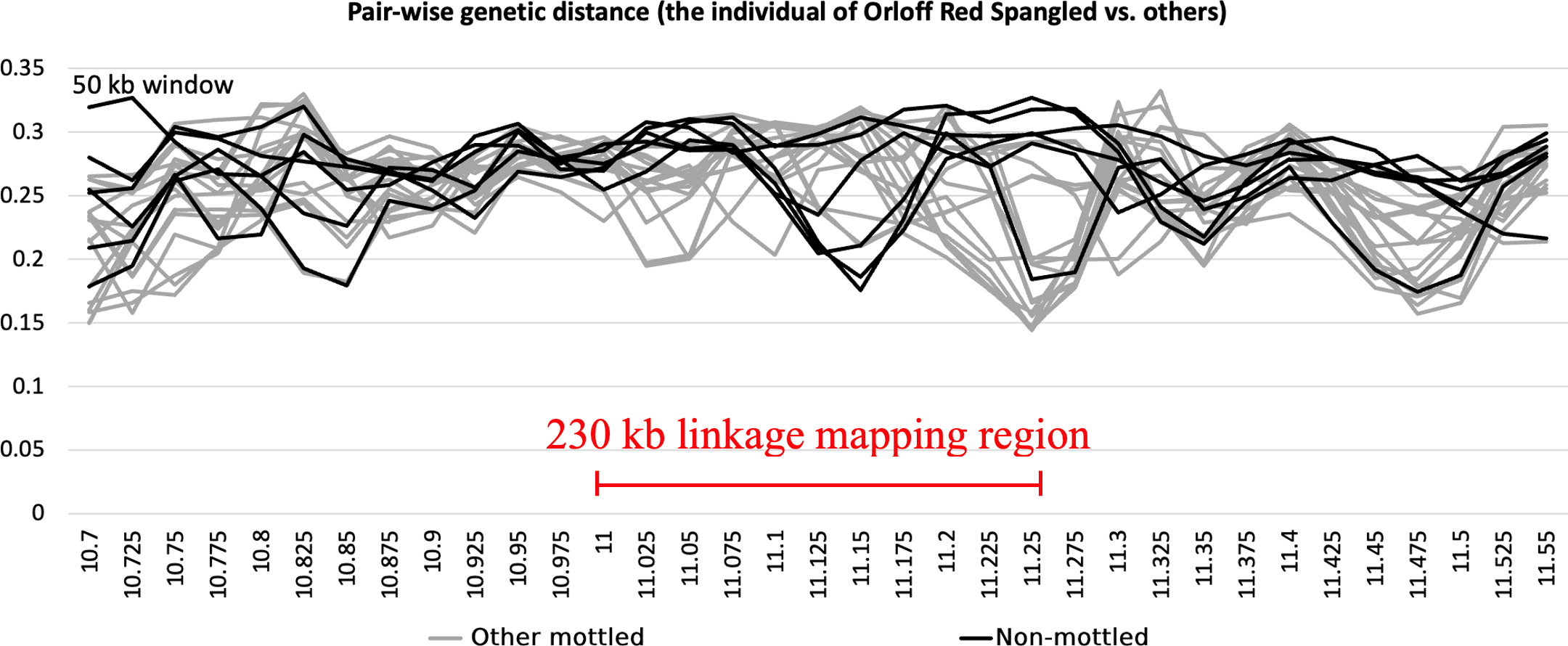

Supplement: Supplementary file 1 — Figure S1: Search of candidate region for MO in Japanese Black Mottled. Plots of pair‐wise genetic distances, based on variable sites only. The orange line indicates the contrast among individual sample of Japanese Black Mottled, grey lines indicate the contrast with other mottled samples (3 Houdan, 2 Gournay, 3 Java, 1 Icelandic native chicken, 3 tricolored Booted Bantam, 3 Orloff Red Spangled, and 1 Aseel Red Mottled), black lines indicate the contrast with non‐mottled samples (2 Black Java, 1 Black Leghorn, and 3 red junglefowl). The X‐axis is the genomic position on chromosome 4 in Mb. The previously reported candidate mutation for Japanese Black Mottled chickens is indicated in red. Figure S2: Search of candidate region for MO in two Orloff Red Spangled samples. Plots of pair‐wise genetic distances, based on variable sites only. The blue line indicates the contrast among individual samples of Orloff Red Spangled, grey lines indicate the contrast with other mottled samples (3 Houdan, 2 Gournay, 3 Java, 1 Icelandic native chicken, 3 tricolored Booted Bantam, 2 Japanese Black Mottled, 1 Orloff Red Spangled, and 1 Aseel Red Mottled), black lines indicate the contrast with non‐mottled samples (2 Black Java, 1 Black Leghorn, and 3 red junglefowl). The X‐axis is the genomic position on chromosome 4 in Mb. The linkage mapping region based on the Mottled Houdan mapping population is indicated in red. Figure S3: Search of candidate region for MO in one Orloff Red Spangled sample. Plots of pair‐wise genetic distances, based on variable sites only, between the individual sample of Orloff Red Spangled and other mottled or non‐mottled samples. Grey lines indicate the contrast with other mottled samples (3 Houdan, 2 Gournay, 3 Java, 1 Icelandic native chicken, 3 tricolored Booted Bantam, 2 Japanese Black Mottled, 2 Orloff Red Spangled, and 1 Aseel Red Mottled), black lines indicate the contrast with non‐mottled samples (2 Black Java, 1 Black Leghorn, and 3 red junglefowl). Th [file AGE-57-0-s001.zip › age70168-sup-0003-FigureS3@Figure S3.png]

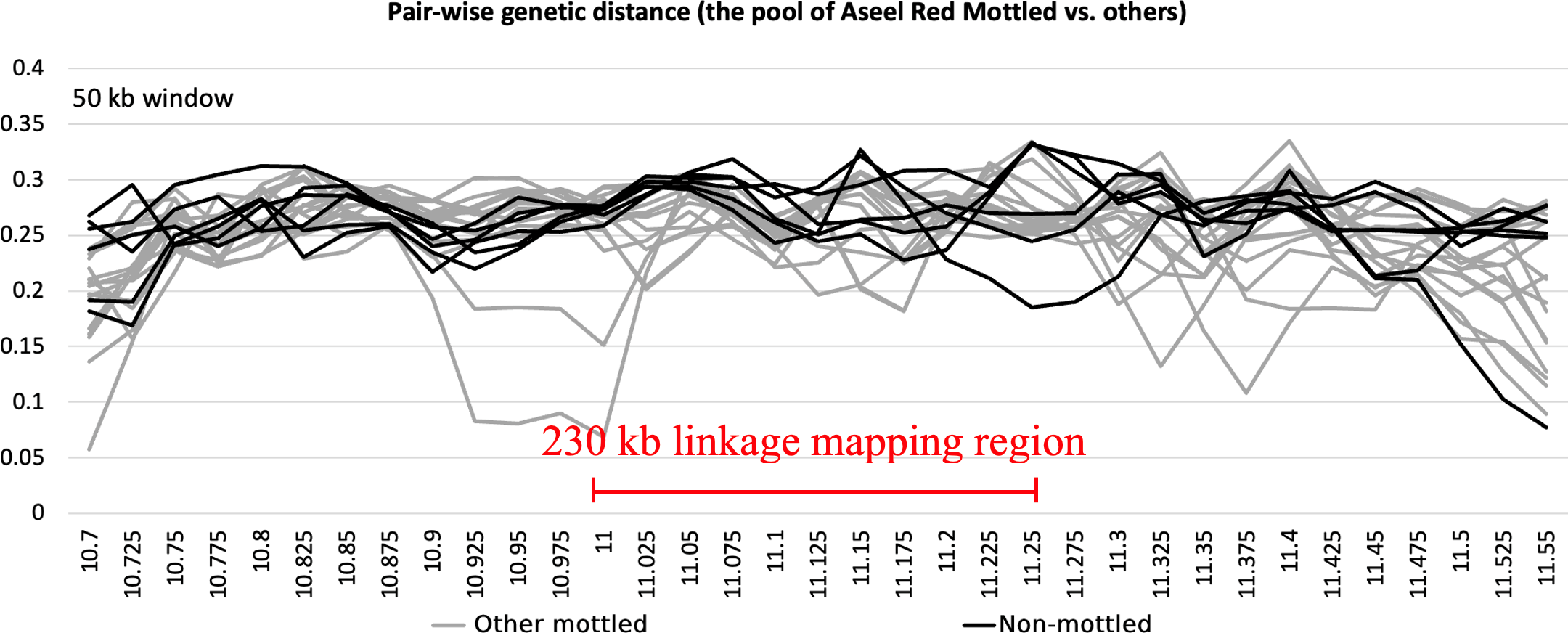

Supplement: Supplementary file 1 — Figure S1: Search of candidate region for MO in Japanese Black Mottled. Plots of pair‐wise genetic distances, based on variable sites only. The orange line indicates the contrast among individual sample of Japanese Black Mottled, grey lines indicate the contrast with other mottled samples (3 Houdan, 2 Gournay, 3 Java, 1 Icelandic native chicken, 3 tricolored Booted Bantam, 3 Orloff Red Spangled, and 1 Aseel Red Mottled), black lines indicate the contrast with non‐mottled samples (2 Black Java, 1 Black Leghorn, and 3 red junglefowl). The X‐axis is the genomic position on chromosome 4 in Mb. The previously reported candidate mutation for Japanese Black Mottled chickens is indicated in red. Figure S2: Search of candidate region for MO in two Orloff Red Spangled samples. Plots of pair‐wise genetic distances, based on variable sites only. The blue line indicates the contrast among individual samples of Orloff Red Spangled, grey lines indicate the contrast with other mottled samples (3 Houdan, 2 Gournay, 3 Java, 1 Icelandic native chicken, 3 tricolored Booted Bantam, 2 Japanese Black Mottled, 1 Orloff Red Spangled, and 1 Aseel Red Mottled), black lines indicate the contrast with non‐mottled samples (2 Black Java, 1 Black Leghorn, and 3 red junglefowl). The X‐axis is the genomic position on chromosome 4 in Mb. The linkage mapping region based on the Mottled Houdan mapping population is indicated in red. Figure S3: Search of candidate region for MO in one Orloff Red Spangled sample. Plots of pair‐wise genetic distances, based on variable sites only, between the individual sample of Orloff Red Spangled and other mottled or non‐mottled samples. Grey lines indicate the contrast with other mottled samples (3 Houdan, 2 Gournay, 3 Java, 1 Icelandic native chicken, 3 tricolored Booted Bantam, 2 Japanese Black Mottled, 2 Orloff Red Spangled, and 1 Aseel Red Mottled), black lines indicate the contrast with non‐mottled samples (2 Black Java, 1 Black Leghorn, and 3 red junglefowl). Th [file AGE-57-0-s001.zip › age70168-sup-0004-FigureS4@Figure S4.png]
